# MTHFR functions as a metabolic checkpoint in NSCLC through SLC25A26-mediated SHMT2 inhibition

**DOI:** 10.1038/s41419-026-08874-z

**Published:** 2026-06-01

**Authors:** Lan li, YiRan Yang, ShiQing Wang, Dan Li, ChuMao Chen, XinMei Mu, JiaXin Wu, Jin Yuan

**Affiliations:** 1 Shanghai Chest Hospital, Shanghai Jiao Tong University School of Medicine, Shanghai, China; 2 Shanghai Key Laboratory of Thoracic Tumor Biotherapy, Shanghai, China; 3 Shanghai Jiao Tong University School of Medicine, Shanghai, China

**Keywords:** Cancer, Cancer

## Abstract

The progression of non-small cell lung cancer (NSCLC) is driven by metabolic plasticity and evasion of apoptotic surveillance, mechanisms that remain incompletely understood. Here, through integrated transcriptomic–metabolomic profiling, molecular interaction mapping, and functional validation, we unveiled a dual regulatory mechanism governed by the MTHFR/SLC25A26 axis that suppresses NSCLC. Clinical cohort analyses revealed concurrent downregulation of MTHFR and SLC25A26 in NSCLC tissues, which strongly correlated with poor prognosis. Mechanistically, MTHFR directly binds and stabilizes SLC25A26, whereas SLC25A26 accelerates SHMT2 degradation via the ubiquitin-proteasome pathway and concurrently suppresses AKT-driven MYB transcriptional activation. This coordinated disruption of serine/one-carbon metabolism and oncogenic signaling significantly inhibited tumor growth in patient-derived xenograft models. Crucially, we identified SLC25A26 as a mitochondrial transporter-ubiquitin adapter hybrid that destabilizes SHMT2 through ubiquitination, whereas its interaction with MTHFR prevents metabolic dysregulation induced by the C677T mutation. Our findings establish the MTHFR/SLC25A26 axis as a master regulator of metabolic–transcriptional crosstalk, providing a therapeutic framework for targeting enzyme stability and kinase signaling in NSCLC treatment.

**Figure:**
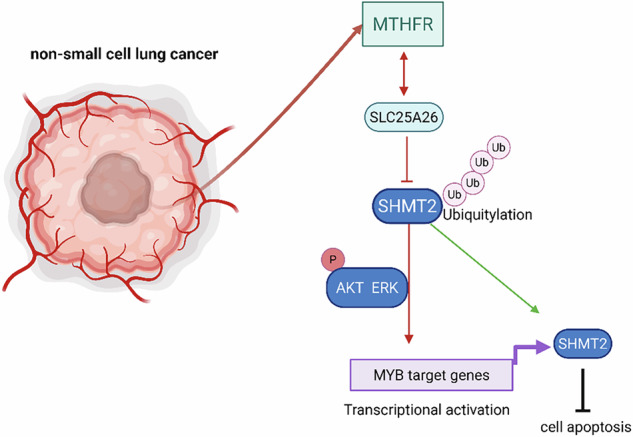
The MTHFR/SLC25A26 axis suppresses NSCLC progression through catalytic activity-dependent stabilization of SLC25A26 by MTHFR, where SLC25A26 functions as a ubiquitin adapter to degrade SHMT2 while concurrently repressing AKT–MYB-driven SHMT2 transcription; clinical downregulation of this axis predicts poor prognosis, and MTHFR overexpression inhibits tumor growth in vivo.

The MTHFR/SLC25A26 axis suppresses NSCLC progression through catalytic activity-dependent stabilization of SLC25A26 by MTHFR, where SLC25A26 functions as a ubiquitin adapter to degrade SHMT2 while concurrently repressing AKT–MYB-driven SHMT2 transcription; clinical downregulation of this axis predicts poor prognosis, and MTHFR overexpression inhibits tumor growth in vivo.

## Introduction

Non-small cell lung cancer (NSCLC), which is responsible for 85% of global lung cancer cases, represents a leading cause of cancer-related mortality, with a five-year survival rate of less than 20%, largely attributable to tumor heterogeneity, metastatic propensity, and chemoresistance [1]. Emerging evidence identifies metabolic reprogramming as a hallmark of NSCLC progression, particularly the hyperactivation of serine and one-carbon metabolism. These pathways sustain rapid proliferation by supplying nucleotide precursors (e.g., dTMP), reducing equivalents (NADPH), and methyl donors such as S-adenosylmethionine (SAM) [2–7]. Mitochondrial serine hydroxymethyltransferase 2 (SHMT2), a rate-limiting enzyme in serine metabolism, catalyses serine-to-glycine conversion while generating 5,10-methylenetetrahydrofolate (5,10-CH2-THF), a critical one-carbon unit for pyrimidine synthesis and redox homeostasis. Clinically, SHMT2 overexpression is correlated with poor prognosis in patients with NSCLC [8]. However, therapeutic targeting of SHMT2 has been hindered by compensatory metabolic pathway activation, necessitating the elucidation of its upstream regulatory network for the development of combination strategies.

Methylenetetrahydrofolate reductase (MTHFR), a pivotal folate metabolic enzyme, irreversibly converts 5,10-CH2-THF to 5-methyltetrahydrofolate (5-CH3-THF), thereby balancing nucleotide biosynthesis and methylation flux [9]. Although epidemiological studies link MTHFR polymorphisms (e.g., C677T) to cancer susceptibility, its role in NSCLC remains controversial. Traditional models suggest that reduced MTHFR activity (e.g., C677T mutation) increases 5,10-CH2-THF levels to promote tumorigenesis [10, 11]. Paradoxically, multiomics analyses of TCGA data revealed significant downregulation of MTHFR mRNA and protein expression in NSCLC tissues, with high MTHFR expression predicting improved survival. This dichotomy suggests that the noncanonical tumor-suppressive functions of MTHFR are potentially mediated through dynamic protein interactions rather than through its enzymatic activity alone.

Recent studies highlight the mitochondrial folate transporter SLC25A26 as a metabolic rheostat that governs mitochondrial–cytosolic folate partitioning and energy metabolism [12]. Beyond its transport function, SLC25A26 modulates AKT signaling—a master regulator of cell survival—by influencing AKT phosphorylation at Thr308 (activating) and Ser473 (stabilizing) [13]. Notably, AKT activation induces MYB-dependent transcriptional upregulation of SHMT2 [14], establishing a feedforward loop between oncogenic signaling and metabolic reprogramming. Nevertheless, whether MTHFR coordinates with SLC25A26 to disrupt this metabolic–transcriptional axis remains unexplored.

On this basis, the present study proposes the following scientific hypothesis: MTHFR inhibits the proliferation of NSCLC and induces apoptosis by directly interacting with SLC25A26, reshaping mitochondrial folate metabolism, and inhibiting the AKT–MYB–SHMT2 signaling axis. To verify this hypothesis, we integrated multiomics analysis, molecular interaction detection, and in vitro and in vivo functional experiments to systematically clarify the dual regulatory mechanism of the MTHFR/SLC25A26 complex on SHMT2 (transcriptional inhibition and ubiquitination-mediated degradation) and evaluated its clinical transformation potential. The findings of this study not only reveal the nonclassical anticancer function of MTHFR in NSCLC but also provide a theoretical basis for the development of new therapeutic strategies based on “metabolic–signaling synergistic targeting”.

## Methods

### Bioinformatics analysis

Pancancer expression profiling of MTHFR was performed using the GEPIA database. Survival analysis was conducted via KM-Plotter. Differentially expressed genes were identified using the DESeq2 package with |log2FC| > 1 and adjusted *p* < 0.05.

### Cellular experiments

All cell lines used in this study (HEK293T, A549, NCI-H520, HBE, HBAS-2B) were purchased from the American Type Culture Collection (ATCC, USA). All cell lines were authenticated by short tandem repeat (STR) profiling, and routinely tested for mycoplasma contamination every 2 months using PCR-based detection. No mycoplasma contamination was detected in any cell line used in the experiments. For genetic manipulation, lentiviral vectors (GeneChem) were used to establish stable overexpression/knockdown cell lines. Transfection efficiency was confirmed by qPCR (ΔΔCt method) and western blotting.

### Lentivirus production and infection

Lentiviruses were produced using a four-plasmid system. HEK293T cells (2.5 × 10^6^) were seeded into 60-mm dishes coated with poly-D-lysine (0.1 mg/mL in sterile water; Sigma‒Aldrich, St. Louis, MO, USA). After 24 h, the cells were serum-starved in 3 mL of Opti-MEM (Gibco, Thermo Fisher Scientific, Waltham, MA, USA) for 16 h. Viral supernatants were harvested 48-h post-transfection, centrifuged at 4000 × *g* for 20 min at 4 °C, and concentrated by resuspending the pellet in phosphate-buffered saline (PBS) at one-tenth of the original volume. Aliquots were stored at −80 °C.

For cell infection, the lentiviral supernatant was mixed 1:1 with fresh antibiotic-free medium and incubated with the target cells for 24 h. Stable cell lines were generated by selecting transduced cells with 2 μg/mL puromycin (Sigma‒Aldrich) for 10 days. These included the knockdown models A549-shscr, A549-shMTHFR, NCI-H520-shscr, and NCI-H520-shMTHFR and the overexpression models A549-EV, A549-MTHFR, H520-EV, and H520-MTHFR.

### Cell viability assay

For the CCK-8 proliferation assay, 5 × 10^3^ cells per well were seeded in a 96-well plate. At 48 and 72 h, CCK-8 reagent (10 μL per well) was added. After the cells were incubated for 2 h, the absorbance was measured at 450 nm using a microplate reader, and the proliferation inhibition rate was calculated.

### Scratch wound healing assay

Cell migration analysis was performed using a standardized scratch wound healing assay.

For cell preparation, NSCLC cells (A549/H520) were seeded in 6-well plates at a density of 5 × 10^5^ cells/well and cultured in RPMI-1640 supplemented with 10% FBS until they reached 90–100% confluence. A sterile 200-μL pipette tip was used to create a linear scratch in the monolayer. The detached cells were removed by washing the cultures twice with PBS. Cells were maintained in serum-free medium, and wound closure was monitored at 0 and 24 h using an inverted phase-contrast microscope (Nikon Eclipse Ti2, 10× objective). Three representative fields per well were imaged. Migration distance was measured using ImageJ software (NIH) by calculating the percentage of wound closure. Data are presented as the mean ± SD from three independent experiments, each performed in triplicate. Statistical significance was determined by two-way ANOVA with the Bonferroni post hoc correction.

### Detection of apoptosis by annexin V-APC/PI double staining

Apoptotic cells were quantified using an Annexin V-APC Apoptosis Detection Kit (BD Biosciences, Cat# 556547) following the manufacturer’s protocol. The cells (1 × 10^6^ cells/group) were harvested 48-h post-transfection, washed twice with cold PBS, and resuspended in 1× binding buffer. Cell suspensions were incubated with 5 μL of Annexin V-APC and 5 μL of propidium iodide (PI) for 15 min at 25 °C in the dark. Samples were analyzed within 1 h using a BD FACSCanto II flow cytometer (BD Biosciences) with excitation at 488 nm. Emission signals were detected at 530 nm (FITC) and 670 nm (PI). A minimum of 10,000 events per sample were recorded.

### Cell cycle analysis via PI staining

Cell cycle distribution was evaluated using PI-based DNA content quantification. The cells were serum-starved for 24 h to synchronize at the G0/G1 phase, after which they were released into complete medium for 12 h. The cells were harvested, washed with PBS, and fixed in 70% ice-cold ethanol at 4 °C overnight. Fixed cells were pelleted, resuspended in PBS supplemented with 50 μg/mL PI (Sigma‒Aldrich, Cat# P4170) and 100 μg/mL RNase A (Thermo Fisher, Cat# EN0531), and incubated at 37 °C for 30 min. The percentages of Annexin V-APC/PI-stained cells were analyzed using FlowJo software.

### RNA extraction and quantitative RT‒PCR

Total RNA was isolated from the cells using TRIzol reagent (Invitrogen) according to the manufacturer’s instructions. One microgram of RNA was reverse transcribed into complementary DNA (cDNA) for use in qPCR. qPCR was performed using the SsoAdvanced SYBR Green Supermix and CFX Connect RT System (Bio-Rad) to examine the expression of MTHFR, with ACTIN as an internal control. The primer pairs used are listed in Supplementary Table 1.

### Western blot

Cells were lysed in ice-cold RIPA buffer (Thermo Fisher Scientific, #89900) supplemented with 1× protease inhibitor cocktail (Roche, #04693159001) for 30 min on ice. The lysates were subsequently centrifuged at 12,000 × *g* for 15 min at 4 °C, after which the proteins in the supernatants were quantified using a BCA Protein Assay Kit (Pierce™, #23225) with bovine serum albumin (BSA) standards. Equal amounts of protein (30 μg per lane) were mixed with 4× Laemmli loading buffer (Bio-Rad, #1610747), denatured at 95 °C for 5 min, and resolved on 10% SDS‒PAGE gels (Bio-Rad Mini-PROTEAN system) at 120 V for 90 min. The separated proteins were transferred to PVDF membranes (Millipore, #IPFL00010) by using a wet transfer system (Trans-Blot® Turbo™, Bio-Rad) at 220 mA for 90 min. The membranes were blocked with 5% nonfat dry milk (Bio-Rad, #1706404) in TBST (20 mM Tris-HCl, 150 mM NaCl, 0.1% Tween-20, pH 7.6) for 1 h at room temperature and subsequently incubated with primary antibodies (see Supplementary Table 2 for antibody details) diluted in blocking buffer overnight at 4 °C with gentle agitation. After three 5-min washes with TBST, the membranes were probed with horseradish peroxidase (HRP)-conjugated goat anti-rabbit or anti-mouse secondary antibodies (Cell Signaling Technology, #7074S/#7076S; 1:5,000 dilution) for 1 h at room temperature. Protein bands were visualized using SuperSignal™ West Pico PLUS Chemiluminescent Substrate (Thermo Fisher Scientific, #34580) and imaged on a ChemiDoc™ MP Imaging System (Bio-Rad).

### Immunoprecipitation

Cells were lysed in ice-cold lysis buffer (25 mM Tris-HCl pH 7.4, 150 mM NaCl, 1 mM EDTA, 1% NP-40, 5% glycerol) supplemented with protease inhibitor cocktail (Roche, #04693132001), 1 mM PMSF (Leagene, #LT0131), and 10 mM N-ethylmaleimide (NEM, for ubiquitination assays; Sigma-Aldrich, #E3876). Following 30 min incubation on ice, lysates were centrifuged at 14,000 × *g* for 30 min at 4 °C. Total protein concentration of supernatants was determined using the BCA Protein Assay Kit (Thermo Fisher Scientific, #23225), with 1% of each lysate retained as the input control. For each immunoprecipitation reaction, 50 μL Protein A/G magnetic beads (Selleck Chemicals, #B23202) were pre-washed with lysis buffer, then incubated with 5 μg of IP-specific antibody (per 500 μg total cellular protein) for 2 h at 4 °C with end-over-end rotation. IP antibodies used were: anti-Flag (Cell Signaling Technology, #8146), anti-HA (Proteintech, #51064-2-AP), anti-StrepII (ABclonal, #AE066), anti-SLC25A26 (Proteintech, #16309-1-AP), and normal rabbit IgG (Cell Signaling Technology, #2729) as the isotype control. Antibody-conjugated beads were washed 3 times with lysis buffer, then incubated with 500 μg total cell lysate overnight at 4 °C with end-over-end rotation. Beads were subsequently washed 6 times with lysis buffer, resuspended in 1× Laemmli loading buffer, and denatured at 95 °C for 10 min.

Immunoprecipitated proteins and input samples were resolved by SDS-PAGE, transferred to PVDF membranes, and analyzed by western blotting. Primary antibodies for detection were: anti-HA (1:1000, Proteintech, #51064-2-AP), anti-StrepII (1:5000, ABclonal, #AE066), anti-Flag (1:1000, Cell Signaling Technology, #8146), anti-SLC25A26 (1:1000, Proteintech, #16309-1-AP), and anti-MTHFR (1:1000, Proteintech, #13063-1-AP).

### Luciferase reporter assay

A luciferase reporter vector, pGL4.27, containing a SHMT2 promoter was constructed to identify the transcriptional activation of SHMT2 by MYB. 293T cells were cotransfected with the indicated doses of the MSCV-strepII-MYB-EGFP (or negative control vector) plasmid and the pGL4.27-SHMT2 promoter vector. Twenty-four hours after transfection, luciferase activity was measured using a luciferase reporting system (GloMax® Multi Instrument). The luciferase activity is defined as the ratio of firefly luciferase units to Renilla luciferase units.

### RNA sequencing

Total RNA was isolated from H520-EV (empty vector control) and MTHFR-OE (MTHFR-overexpressing) NSCLC cells using TRIzol® Reagent (Invitrogen, #15596026) following the manufacturer’s protocol. Afterwards, library construction and sequencing were carried out. Raw data analysis was performed, followed by alignment and quantification to the human reference genome (GRCh38) using HISAT2 (v2.2.1). Feature Counts (v2.0.3) was used to calculate the gene expression levels (TPM values). DESeq2 (v1.34.0) was used to screen for differentially expressed genes (|log2FC |>1, FDR < 0.05). For functional enrichment analysis, clusterProfiler (v4.4.4) was used to perform GO and KEGG pathway enrichment analyses (*p* < 0.05). Differential genes were further validated by qRT‒PCR using H520-EV/MTHFR-OE RNA to identify target genes.

### Immunofluorescence and confocal microscopy

A549/H520-EV (empty vector control) and A549/H520-SLC25A26-OE (MTHFR-overexpressing) cells were seeded in 12-well plates (6 × 10^5^ cells/well) containing sterile round glass coverslips and cultured for 24 h. The cells were fixed with 4% paraformaldehyde for 20 min at room temperature, washed three times with phosphate-buffered saline (PBS), and permeabilized with 0.1% Triton X-100 in PBS for 10 min. After three additional PBS washes, nonspecific binding sites were blocked with 3% BSA in PBS for 2 h at room temperature.

The cells were incubated overnight at 4 °C with a primary antibody against SHMT2 (1:100, Cat# A21803, ABclonal, Wuhan, China), followed by three washes with 0.05% PBST (PBS containing 0.05% Tween 20). A fluorescein-conjugated secondary antibody (1:500, Cat# BA1032, Boster Biological Technology, Pleasanton, CA, USA) was applied for 1 h at room temperature. After three washes with PBST, the nuclei were counterstained with 0.1 μg/mL DAPI (Sigma‒Aldrich, St. Louis, MO, USA) for 5 min. Coverslips were mounted onto glass slides using antifade mounting medium and imaged with a Nikon ECLIPSE 80i laser scanning confocal microscope (Nikon Instruments, Tokyo, Japan).

### Molecular docking simulation

The crystal structures of human wild-type (WT) MTHFR (PDB ID: 6FCF) and SLC25A26 (PDB ID: 6G13) were retrieved from the RCSB Protein Data Bank. The MTHFR A222V mutant (corresponding to the C677T polymorphism) was constructed based on the optimized WT-MTHFR structure. All protein structures were pre-processed using AutoDock Tools 1.5.7, including removal of water molecules, addition of polar hydrogens, and assignment of Gasteiger charges.

Protein-protein docking was performed using AutoDock Vina 1.2.3. The grid box was defined to cover the entire potential binding interface between MTHFR and SLC25A26, with an exhaustiveness value of 32. The optimal binding conformation was screened by the lowest binding free energy and cluster analysis. The binding interface, key interacting residues, and interaction types were analyzed and visualized using PyMOL 2.5.

### Ubiquitination assay

To investigate SHMT2 ubiquitination, HEK293T cells were transiently cotransfected with plasmids encoding HA-tagged ubiquitin (1:1000, 51064-2-AP, Proteintech) and Flag-tagged SHMT2 (1:1000, Cat# 8146, Cell Signaling) using Lipofectamine 3000 (Thermo Fisher, #L3000015) at a 1:3 DNA-to-reagent ratio. At 24-h post-transfection, the cells were treated with 10 μM MG132 (MedChemExpress, #HY-13259) in DMSO for 6 h to stabilize ubiquitinated proteins.

### Untargeted metabolomics

Metabolomic analysis was performed using ultra-performance liquid chromatography‒mass spectrometry (UPLC‒MS). Cellular metabolites were extracted from cell lysates with 80% methanol, followed by chromatographic separation using an ACQUITY UPLC BEH C18 column (Waters). Mass spectrometry detection was performed on a QE Plus instrument (Thermo Scientific) operating in both positive and negative ion modes. After data were normalized, multivariate statistical analyses, including principal component analysis and partial least squares-discriminant analysis, were performed using MetaboAnalyst 5.0. Differentially abundant metabolites were identified based on variable importance in projection scores >1 and statistical significance (*p* < 0.05).

### U-^13^C-serine isotope tracing assay

A549 and NCI-H520 cells were incubated in serine/glycine-free RPMI 1640 medium supplemented with 0.4 mM U-^13^C3-L-serine (Cambridge Isotope Laboratories, #CLM-1574-PK) for 12 h, with 3 independent biological replicates per group. Intracellular metabolites were extracted using pre-chilled methanol:acetonitrile:water (40:40:20, v/v/v) with 2-chloro-L-phenylalanine as an internal standard. Metabolite detection was performed on an Ultimate 3000 UHPLC system coupled to a Q Exactive HF-X Orbitrap mass spectrometer (Thermo Fisher Scientific) with a Waters ACQUITY BEH Amide column, operated in positive/negative ion switching mode. Target isotopically labeled metabolites were identified by accurate mass and MS² spectra matching to authentic standards. The ¹³C isotope incorporation rate was calculated as the percentage of labeled metabolite peak area relative to the total peak area of all isotopic forms of the corresponding metabolite. Statistical significance was determined by unpaired two-tailed Student’s *t* test.

### Haematoxylin and eosin and immunohistochemistry

Tissue samples were fixed in 10% neutral-buffered formalin for 24 h, embedded in paraffin, and sectioned at a thickness of 5 μm. The sections were deparaffinized in xylene, rehydrated through a graded ethanol series, and stained with Harris haematoxylin (Cat# G1004, Servicebio, Wuhan, China) for 5 min, followed by eosin Y (Cat# G1100, Servicebio) for 1 min. The slides were dehydrated, cleared in xylene, and mounted with neutral resin. For antigen retrieval, deparaffinized sections were heated in 10 mM citrate buffer (pH 6.0) in a microwave (95 °C, 10 min) and cooled to room temperature. Endogenous peroxidase activity was quenched with 3% hydrogen peroxide (H₂O₂) for 15 min. After three washes in PBS, the sections were blocked with 5% goat serum (Cat# 42450, SparkJade, Qingdao, China) at 25 °C for 1 h. A primary antibody against Ki67 (1:800 dilution, Cat# 9129S, Cell Signaling Technology, Danvers, MA, USA) was applied overnight at 4 °C. The sections were incubated with an HRP-conjugated goat anti-rabbit secondary antibody (1:1000 dilution, Cat# abs20028, Absin, Shanghai, China) for 2 h at 37 °C. Signals were detected using 3,3’-diaminobenzidine (DAB; Cat# 42450, SparkJade) for 3 min, followed by counterstaining with haematoxylin (Cat# BA-4041, Baso, Zhuhai, China). The stained sections were imaged using a Nikon Eclipse E100 brightfield microscope (Nikon, Tokyo, Japan) at 40× magnification.

### In vivo xenograft studies

For the NCI-H520 xenograft model, male BALB/c nude mice (6–8 weeks old) were subcutaneously injected into the dorsal flank with the following: Control group: NCI-H520-EV cells (empty vector, 1 × 10^7^ cells/mouse; *n* = 5); MTHFR-OE group: NCI-H520-MTHFR-OE cells (1 × 10^7^ cells/mouse; *n* = 5); Rescue groups: MTHFR-OE + SHMT2-OE: NCI-H520-MTHFR-OE cells coexpressing SHMT2 (1 × 10^7^ cells/mouse; *n* = 5); MTHFR-OE + shSLC25A26 group: NCI-H520-MTHFR-OE cells with SLC25A26 knockdown (1 × 10^7^ cells/mouse; *n* = 5). The mice were monitored for 28 days post- injection. Tumors were excised, photographed, and weighed at the experimental endpoint.

### Statistical analysis

All statistical analyses were performed using GraphPad Prism 8.0 (GraphPad Software, USA), with a two-sided significance threshold set at *P* < 0.05 for all tests. Quantitative data are presented as mean ± standard deviation (SD), with the number of independent biological replicates (n) explicitly stated in each figure legend. Technical replicates were used only for assay consistency validation and were not included in the final statistical analyses. Normality of data distribution and homogeneity of variances were confirmed via the Shapiro–Wilk test and Levene’s test, respectively, prior to all parametric statistical tests.Unpaired two-tailed Student’s *t* test: Used for comparisons between two independent experimental groups (e.g., EV vs MTHFR-OE, Scramble vs shMTHFR). One-way ANOVA followed by Student–Newman–Keuls (SNK) post-hoc test: Used for comparisons among three or more independent groups. Significance levels were uniformly defined as *P < 0.05, **P < 0.01, and ***P < 0.001, with non-significant results marked as “ns”. The specific statistical test, number of biological replicates, and significance thresholds are explicitly detailed in the legend of every figure in the manuscript.

## Results

### MTHFR downregulation in NSCLC is correlated with adverse clinical outcomes

**Fig. 1:**
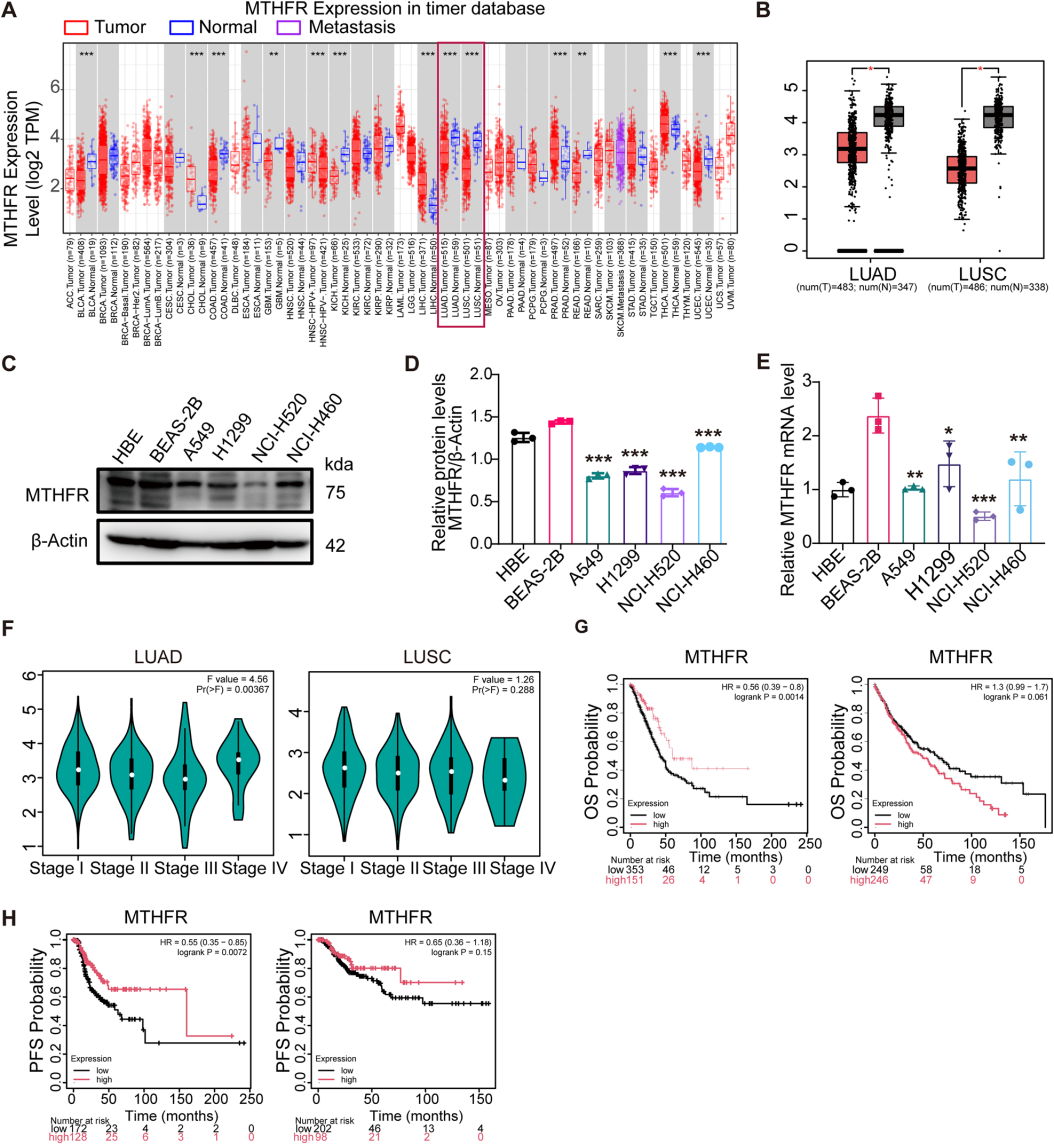
Downregulation of MTHFR expression correlates with poor clinical prognosis in non-small cell lung cancer (NSCLC). **A** Pancancer analysis of MTHFR expression levels across tumor types using the TIMER 2.0 database. **B** UALCAN database analysis revealed significantly downregulated MTHFR mRNA expression in NSCLC tissues compared with adjacent normal tissues. **C**, **D** Immunoblotting analysis of MTHFR protein levels in two human lung fibroblast lines (HBE and HBAS-2B) and four NSCLC cell lines (A549, H1299, H520, and H460). **E** Quantitative RT‒PCR validation of MTHFR mRNA expression in the same cell models. **F** Association of MTHFR expression levels with clinical stage in lung squamous cell carcinoma (LUSC) and lung adenocarcinoma (LUAD) cohorts from the TCGA database. **G**, **H** Kaplan‒Meier survival curves for overall survival (OS) and progression-free survival (PFS) based on MTHFR expression levels in NSCLC tissues generated using the Kaplan‒Meier plotter database. Means ± SEM, *P < 0.05; **P < 0.01; ***P < 0.001; *t*-test.

To systematically investigate the clinical relevance of MTHFR in non-small cell lung cancer (NSCLC), we performed integrated multiomics analyses across multiple cohorts. Pancancer expression profiling using the TIMER 2.0 and TCGA datasets revealed significant MTHFR downregulation in malignant versus normal tissues (Fig. 1A). Subsequent validation in NSCLC subtypes through GEPIA (Gene Expression Profiling Interactive Analysis) demonstrated consistent reductions in MTHFR expression in both lung adenocarcinoma (LUAD) and lung squamous cell carcinoma (LUSC) compared with adjacent normal tissues (Fig. 1B).

At the cellular level, multiplatform validation showed consistent expression patterns: qRT‒PCR and western blot analyses revealed higher MTHFR expression in immortalized bronchial epithelial cells (HBE and HBAS-2B) than in NSCLC cell lines (Fig. 1C–E). Based on this molecular stratification, we established A549 (LUAD) and H520 (LUSC) cells as representative low-MTHFR-expressing models for functional studies.

Clinical correlation analysis revealed that MTHFR expression differed significantly across early, intermediate, and advanced stages of LUAD, whereas no significant differences were observed among these stages in LUSC (Fig. 1F). Furthermore, analysis of TCGA data indicated that high MTHFR expression was associated with improved overall survival (OS) and progression-free survival (PFS) in patients with LUAD, whereas only a nonsignificant trend was observed in patients with LUSC (Fig. 1G, H). This prognostic pattern exhibited pancancer relevance, as evidenced by Cox proportional hazards models showing consistent reduction in OS across 8 additional tumor types (Supplementary Fig. 1).

### MTHFR overexpression suppresses proliferation and induces apoptosis in NSCLC

**Fig. 2:**
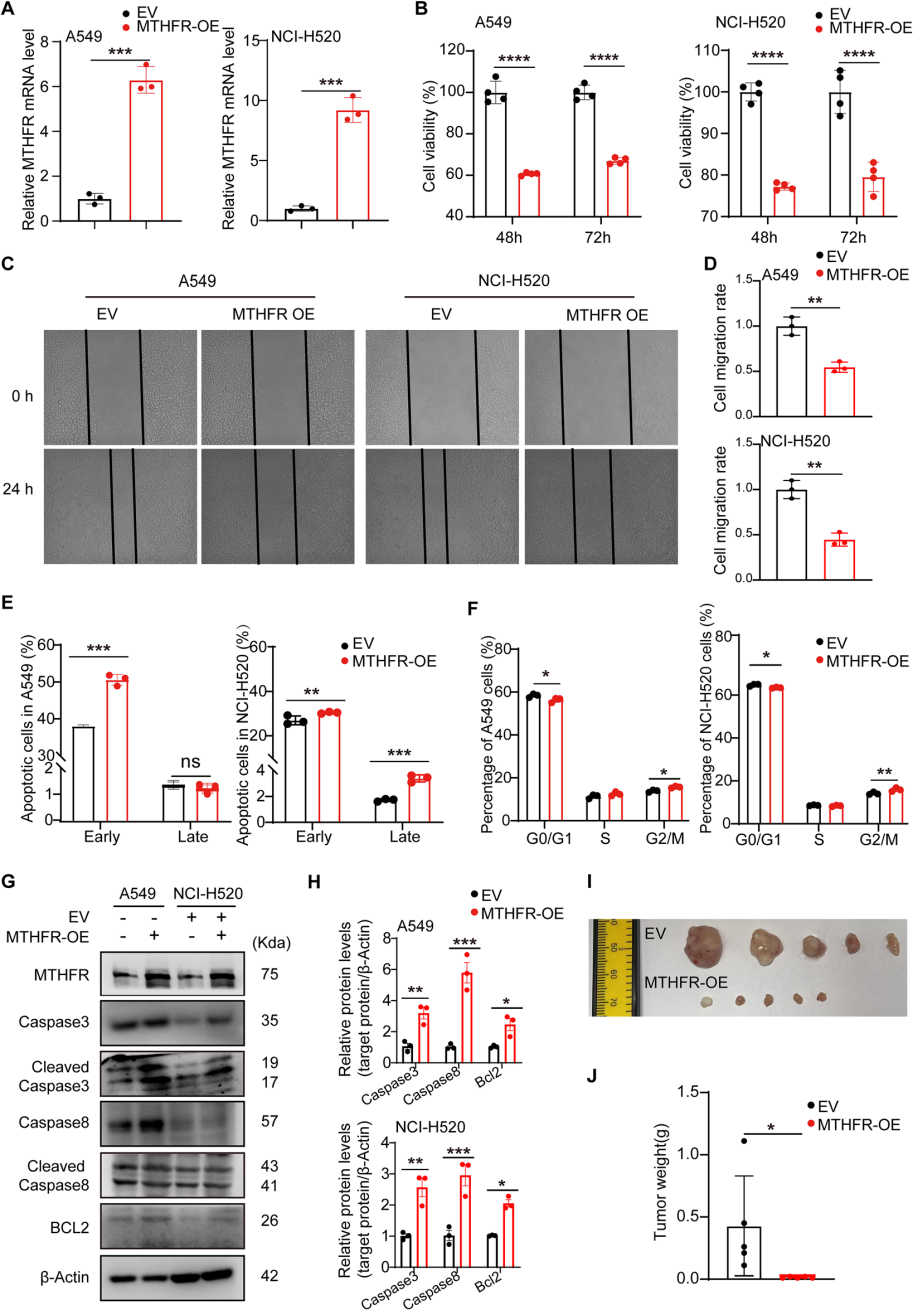
MTHFR overexpression suppresses proliferation and induces apoptosis in NSCLC. **A** RT‒qPCR validation of MTHFR overexpression in A549 (LUAD) and NCI-H520 (LUSC) cell lines. **B** CCK-8 proliferation assays comparing empty vector (EV)-transfected and MTHFR-overexpressing (MTHFR-OE) cells at 48 h and 72 h post-transduction. **C**, **D** Wound-healing assays demonstrating impaired migratory capacity in MTHFR-OE cells at 24 h. Representative images (**C**) and quantitative analysis (**D**) of wound closure rates. **E** Flow cytometry analysis of apoptosis in EV and MTHFR-OE cells, showing the percentage of early apoptotic (annexin V+/PI−) and late apoptotic (annexin V+/PI+) cells. **F** Cell cycle distribution analysis by flow cytometry, revealing G1 phase depletion and G2/M arrest in MTHFR-OE cells. **G** MTHFR overexpression promotes pro-apoptotic signaling. Immunoblot showing the protein levels of cleaved caspase-3, caspase-8, and Bcl-2 in control and MTHFR-OE cells. β-actin was used as a loading control. **H** Densitometric quantification of protein levels normalized to that of β-actin (**P < 0.01, ***P < 0.001). **I**, **J** In vivo tumor growth suppression by MTHFR overexpression: Representative images of subcutaneous xenografts (**I**) and tumor weights (**J**) at the endpoint (*n* = 5 mice/group; *P < 0.05). Means ± SEM, *P < 0.05; **P < 0.01; ***P < 0.001; *t*-test.

Stable MTHFR-overexpressing A549 (LUAD) and H520 (LUSC) cell lines were established via lentiviral transduction, with qRT‒PCR confirming the 5- to 10-fold upregulation of MTHFR mRNA expression compared with that in vector controls (Fig. 2A). Functional characterization revealed potent antiproliferative effects: CCK-8 assays demonstrated a reduction in viability at 48 h and 72 h post-transduction in MTHFR-overexpressing cells compared with that in control cells (Fig. 2B). Conversely, MTHFR knockdown significantly increased proliferation rates (Supplementary Fig. S2C, D). Wound-healing assays revealed impaired migration capacity in the overexpression groups at 24 h (Fig. 2C, D).

Apoptotic profiling via annexin V/PI flow cytometry revealed cell type-dependent effects: H520 cells exhibited 3.1-fold increases in early (annexin V+/PI−) and late (annexin V+/PI+) apoptosis, whereas A549 cells showed a selective increase in early apoptosis without significant late-stage changes (Fig. 2E, Supplementary Fig. S2A). Knockdown reciprocally reduced apoptosis (Supplementary Fig. S2E, F). Cell cycle analysis revealed G1 phase depletion and G2/M accumulation in MTHFR-overexpressing cells, indicating that proliferation was blocked (Fig. 2F, Supplementary Fig. S2B).

Additionally, we assessed the expression of apoptosis-related proteins by western blotting. The results demonstrated that overexpression of MTHFR led to increases in both the full-length and cleaved forms of the apoptotic proteins caspase-3 and caspase-8 but concurrently resulted in a corresponding decrease in the antiapoptotic protein BCL-2. These findings indicate that MTHFR suppresses cell proliferation by promoting apoptosis in NSCLC (Fig. 2G, H). In vivo validation using subcutaneous xenograft models revealed a reduction in tumor volume and a decrease in weight in MTHFR-overexpressing H520 tumors compared with controls at the endpoint (Fig. 2I, J).

### SLC25A26, a downstream target of MTHFR, inhibits proliferation and apoptosis in NSCLC

**Fig. 3:**
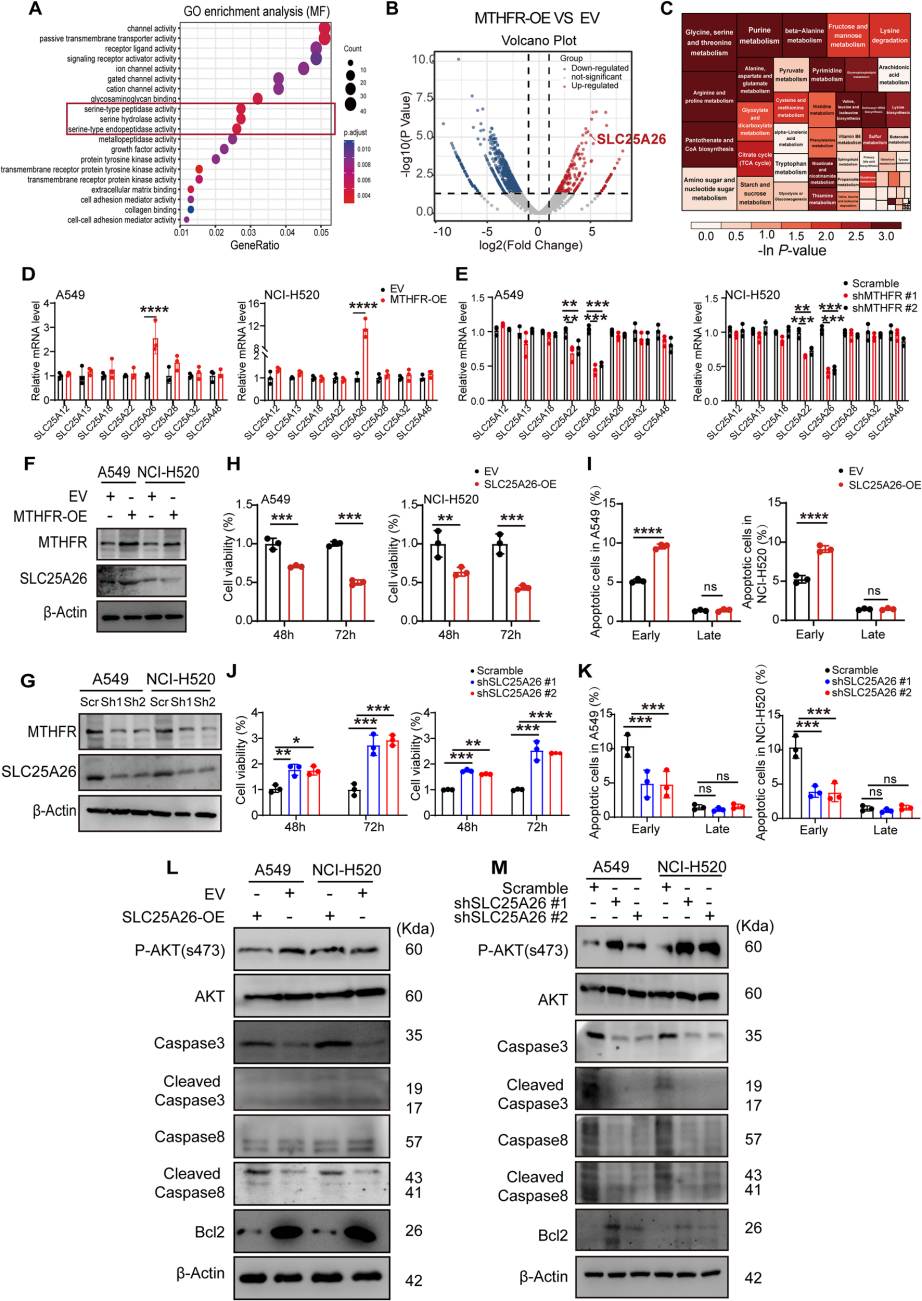
SLC25A26 acts as a downstream effector of MTHFR to inhibit proliferation and promote apoptosis. **A** Gene Ontology (GO) molecular function (MF) enrichment analysis of MTHFR-regulated targets, highlighting enhanced serine metabolic processes (red box). **B** Volcano plot of RNA-seq data from H520 cells overexpressing MTHFR versus controls (|log2-fold change (FC)| >1, *P* < 0.05), identifying SLC25A26 as the top upregulated gene. **C** Metabolomic pathway enrichment analysis (treemap) comparing metabolic profiles of MTHFR-overexpressing and control H520 cells. **D**, **E** MTHFR specifically regulates SLC25A26 expression. qPCR analysis of SLC25A26 and other SLC25A family transporters in cells following MTHFR overexpression (OE) or knockdown (KD). **F**, **G** MTHFR positively regulates SLC25A26 protein levels. Representative immunoblots of SLC25A26 expression in control, MTHFR-overexpressing (**F**), and MTHFR-knockdown (**G**) cells. β-Actin serves as a loading control. **H**–**K** SLC25A26 inhibits proliferation and induces apoptosis in NSCLC cells. CCK-8 proliferation assays in A549 and NCI-H520 cells following SLC25A26 overexpression (**H**) or knockdown (**J**). Apoptosis assays in A549 and NCI-H520 cells following SLC25A26 overexpression (**I**) or knockdown (**K**). **L**, **M** SLC25A26 modulates the apoptotic pathway and AKT phosphorylation. **L** Immunoblot analysis of apoptosis-related proteins (full-length and cleaved caspase-3/8, BCL-2) and p-AKT (Ser473) in control and SLC25A26-overexpressing cells. **M** Immunoblot analysis of the same proteins in control and SLC25A26-knockdown cells. β-Actin was used as a loading control. Means ± SEM, *P < 0.05; **P < 0.01; ***P < 0.001; *t*-test.

To investigate how MTHFR modulates apoptosis and proliferation in NSCLC, we conducted transcriptomic profiling of H520 cell lines and compared the control and MTHFR-overexpressing groups. A volcano plot (|log2FC| > 1, *p* < 0.05) revealed significant upregulation of SLC25A26 mRNA upon MTHFR overexpression (Fig. 3B). SLC25A26, a mitochondrial SAM transporter, indirectly regulates one-carbon unit metabolism derived from serine by modulating mitochondrial SAM levels. Gene Ontology (GO) analysis demonstrated enhanced serine metabolism (Fig. 3A), whereas KEGG pathway analysis revealed enrichment of the AKT signaling pathway (Supplementary Fig. 3A). Further in vitro validation studies demonstrated that overexpression of MTHFR inhibited the phosphorylation of AKT (S473) in NSCLC cell lines (Supplementary Fig. 3B), whereas knockdown of MTHFR increased AKT (S473) phosphorylation in NSCLC cell lines (Supplementary Fig. 3C). Given the pivotal role of MTHFR in folate metabolism, we performed complementary metabolomics profiling, which confirmed elevated serine levels (Fig. 3C, Supplementary Fig. 3D–F).

Notably, the mitochondrial carrier family member SLC25A26 specifically mediates the transport of SAM from the cytosol into mitochondria and the export of S-adenosylhomocysteine to the cytosol. qPCR analysis revealed that overexpression of MTHFR increased SLC25A26 mRNA levels, whereas knockdown of MTHFR decreased them. In contrast, no significant changes in the mRNA levels of other SLC25A transporters (SLC25A12, SLC25A13, SLC25A18, SLC25A22, SLC25A28, SLC25A32, and SLC25A48) were detected (Fig. 3D, E). Consistent with these findings, at the protein level, overexpression of MTHFR led to elevated SLC25A26 expression, whereas knockdown of MTHFR resulted in reduced SLC25A26 levels (Fig. 3F, G). Given its functional role as a solute carrier protein that facilitates SAM transport and its downregulation in NSCLC, which is correlated with poor prognosis, we hypothesized that SLC25A26 is a downstream effector of MTHFR (Supplementary Fig. 3G–J).

To further investigate the impact of SLC25A26 on NSCLC, we overexpressed or knocked down SLC25A26 in A549 and NCI-H520 cells. CCK-8 assays demonstrated that overexpression of SLC25A26 suppressed proliferation in both A549 and NCI-H520 cells (Fig. 3H), whereas knockdown of SLC25A26 promoted proliferation (Fig. 3J). The results of apoptosis assays revealed that SLC25A26 overexpression promoted apoptosis in A549 and NCI-H520 cells (Fig. 3I), whereas SLC25A26 knockdown inhibited apoptosis (Fig. 3K). Furthermore, western blot analysis yielded consistent results. Overexpression of SLC25A26 increased the protein levels of both full-length and cleaved caspase-3/8 but decreased the level of the antiapoptotic protein BCL-2 and reduced the phosphorylation of AKT at Ser473. Conversely, knockdown of SLC25A26 decreased the levels of full-length and cleaved caspase-3/8, increased BCL-2 expression, and increased AKT phosphorylation at Ser473. Collectively, these findings establish SLC25A26 as a tumor-suppressive mediator downstream of MTHFR, inhibiting NSCLC proliferation and promoting apoptosis through SAM-dependent metabolic regulation and AKT signaling modulation.

### A catalytic activity-dependent interaction between MTHFR and SLC25A26 governs apoptotic resistance

**Fig. 4:**
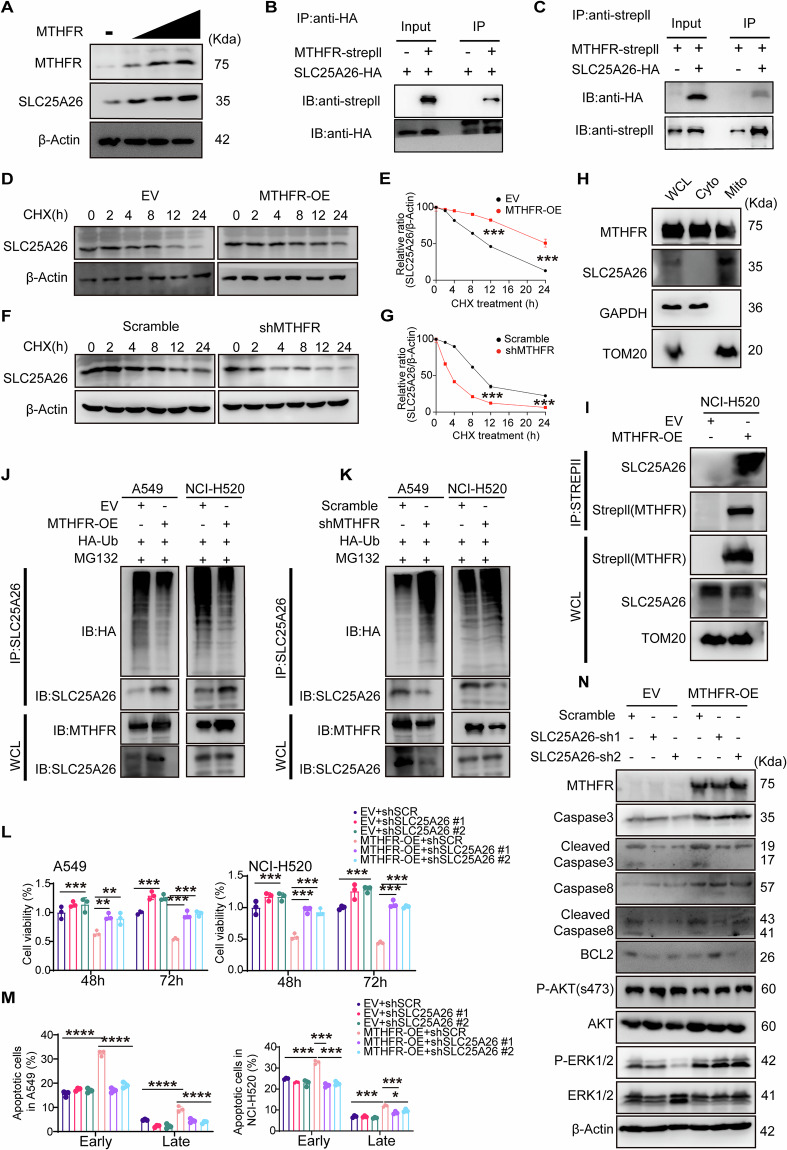
MTHFR interacts with and stabilizes SLC25A26 to exert tumor‑suppressive functions in NSCLC. **A** Western blot showing that MTHFR overexpression increases SLC25A26 protein abundance in 293T cells. **B**, **C** Reciprocal co‑immunoprecipitation assays confirming the physical interaction between StrepII‑MTHFR and HA‑SLC25A26 in 293T cells. **D**, **E** SLC25A26 protein stability upon cycloheximide treatment in MTHFR‑overexpressing H520 cells. Representative blots (**D**) and quantification (**E**). **F**, **G** SLC25A26 protein stability upon cycloheximide treatment in MTHFR‑knockdown H520 cells. Representative blots (**F**) and quantification (**G**). **H** Subcellular fractionation showing the distribution of MTHFR and SLC25A26 in whole‑cell lysates, cytoplasm, and mitochondria. **I** Co‑immunoprecipitation using isolated mitochondria verifying the interaction between endogenous MTHFR and SLC25A26. In vivo ubiquitination assays showing that MTHFR overexpression inhibits (**J**), whereas MTHFR knockdown promotes (**K**), SLC25A26 polyubiquitination. Rescue experiments showing that SLC25A26 knockdown reverses the MTHFR‑mediated inhibition of cell viability (**L**) and promotion of apoptosis (**M**) in H520 cells. **N** Western blot showing that SLC25A26 depletion restores p‑AKT, p‑ERK1/2, and Bcl‑2 levels and reduces cleaved caspase‑3 and caspase‑8 in MTHFR‑overexpressing cells. Means ± SEM, *P < 0.05; **P < 0.01; ***P < 0.001; *t*-test.

We next delineated the molecular mechanism underlying the tumor‑suppressive function of MTHFR in NSCLC via targeting SLC25A26. Overexpression of MTHFR markedly increased SLC25A26 protein abundance in 293T cells (Fig. 4A). Reciprocal co‑immunoprecipitation assays verified a direct physical interaction between MTHFR and SLC25A26 in 293T cells (Fig. 4B, C), which was further supported by their prominent co‑localization (Supplementary Fig. S4A).

We subsequently investigated whether MTHFR modulates SLC25A26 protein stability via cycloheximide (CHX) chase assays. Bidirectional validation showed that MTHFR overexpression significantly slowed the degradation rate of SLC25A26 and prolonged its protein half-life (Fig. 4D, E), whereas knockdown of endogenous MTHFR markedly accelerated SLC25A26 degradation and shortened its half-life in NSCLC cells (Fig. 4F, G). These results demonstrated that MTHFR is both necessary and sufficient to stabilize SLC25A26 protein. Given that SLC25A26 is a canonical mitochondrial transporter, we performed subcellular fractionation assays to clarify the subcellular context of the MTHFR-SLC25A26 interaction. The results showed that MTHFR was distributed in whole-cell lysate, cytoplasmic and mitochondrial fractions, while SLC25A26 was predominantly detected in whole-cell lysate and the mitochondrial compartment (Fig. 4H). Co-IP assays using isolated mitochondria further confirmed that MTHFR directly interacts with SLC25A26 within the mitochondrial compartment, which is consistent with the functional localization of SLC25A26 (Fig. 4I).

To elucidate the mechanism underlying MTHFR-mediated SLC25A26 stabilization, we performed in vivo ubiquitination assays. The results showed that MTHFR overexpression significantly inhibited the polyubiquitination of SLC25A26, while MTHFR knockdown markedly enhanced SLC25A26 polyubiquitination (Fig. 4J, K), revealing that MTHFR stabilizes SLC25A26 by suppressing its ubiquitin-dependent proteasomal degradation. We then performed rescue experiments to verify that SLC25A26 is the essential downstream effector of MTHFR in NSCLC. CCK-8 viability assays and flow cytometry apoptosis analysis showed that SLC25A26 knockdown significantly reversed the anti-proliferative and pro-apoptotic effects induced by MTHFR overexpression (Fig. 4L, M, Supplementary Fig. 4K). Consistent with the phenotypic rescue, western blot analysis demonstrated that SLC25A26 depletion fully restored the activation of pro-survival signaling pathways in MTHFR-overexpressing cells, as evidenced by upregulated phosphorylation of AKT (Ser473) and ERK1/2 (Thr202/Tyr204), increased anti-apoptotic protein Bcl-2 expression, and reduced cleavage of pro-apoptotic caspase-3 and caspase-8 (Fig. 4N).

Given the well-documented clinical relevance of the MTHFR C677T polymorphism, which causes an alanine-to-valine substitution at residue 222 (A222V) and abolishes the catalytic activity of MTHFR, we generated this catalytically inactive MTHFR-C677T mutant to further validate the structural and functional dependence of the MTHFR-SLC25A26 axis (Supplementary Fig. 4B). Protein-protein molecular docking simulation showed that wild-type MTHFR forms a stable binding complex with SLC25A26 via a well-defined hydrophobic interaction interface (Supplementary Fig. 4C), while the A222V substitution induced significant conformational rearrangement of the binding interface and disrupted the stable interaction between MTHFR and SLC25A26 (Supplementary Fig. 4D). In line with the structural simulation results, reciprocal co-IP assays confirmed that the physical interaction between MTHFR and SLC25A26 was completely abrogated by the C677T mutation (Supplementary Fig. 4E). Functional assays further revealed that overexpression of the MTHFR-C677T mutant exerted an opposite tumor-promoting phenotype to wild-type MTHFR: it significantly promoted proliferation in both A549 and NCI-H520 NSCLC cells in a time-dependent manner (Supplementary Fig. 4H), and significantly inhibited apoptosis in NSCLC cells (Supplementary Fig. 4F, G). In vivo subcutaneous xenograft models further confirmed that MTHFR overexpression suppressed tumor growth, while the MTHFR‑C677T mutant abolished this effect and restored tumor growth (Supplementary Fig. 4I, J). Collectively, these data demonstrate that MTHFR interacts with and stabilizes SLC25A26 in a catalytic activity‑dependent manner, thereby restraining AKT/ERK pro‑survival signaling and exerting potent tumor‑suppressive functions in NSCLC.

### MTHFR regulates one-carbon metabolism via SHMT2 suppression

**Fig. 5:**
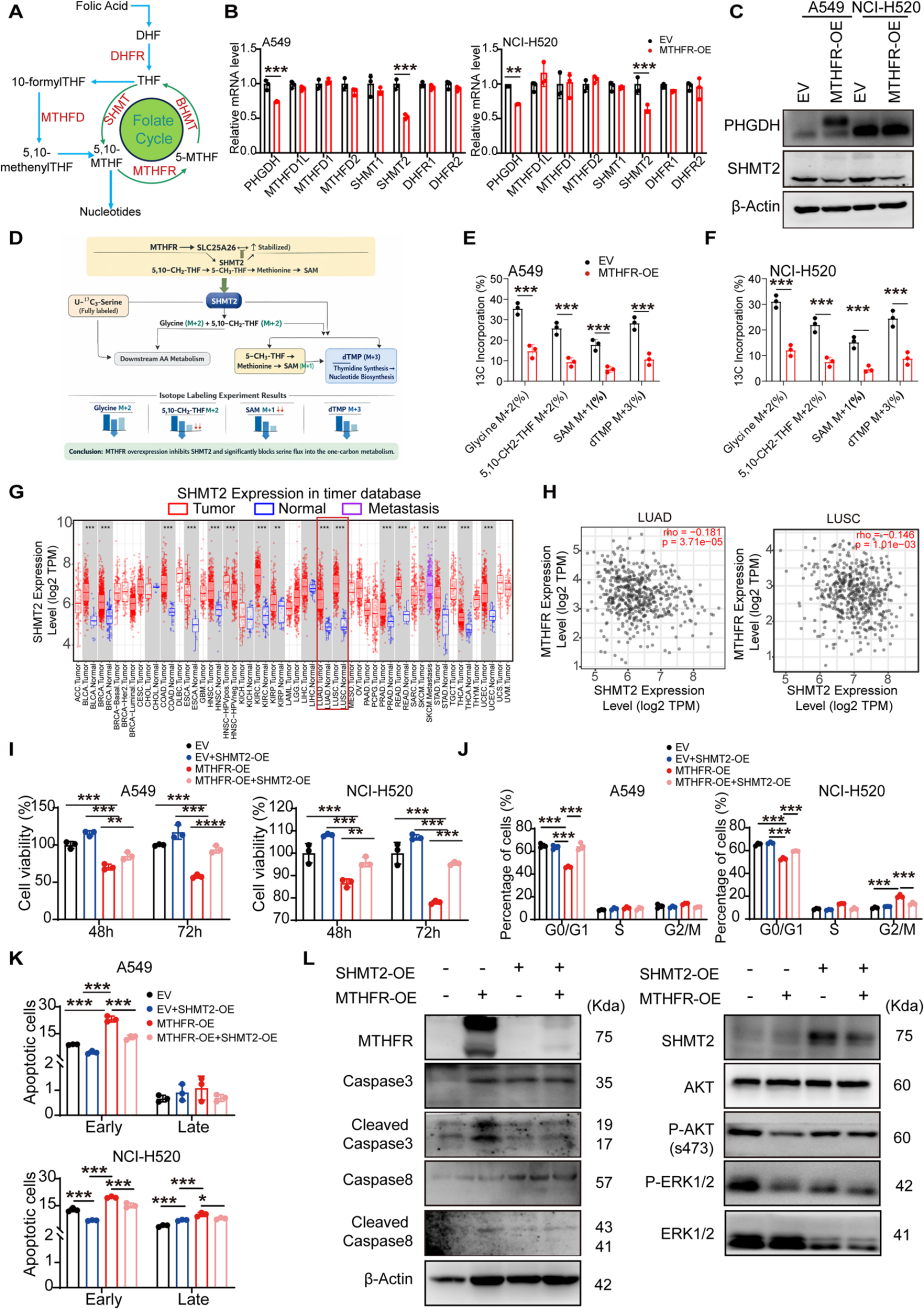
MTHFR suppresses NSCLC tumor progression via transcriptional repression of SHMT2 and perturbation of one-carbon metabolism. **A** Schematic diagram of the folate-driven one-carbon metabolism pathway, highlighting the folate cycle and methionine regeneration. **B** qPCR analysis of key one-carbon metabolic genes in control and MTHFR-overexpressing (MTHFR-OE) cells. MTHFR overexpression significantly downregulates SHMT2 and PHGDH mRNA levels. **C** Immunoblot analysis of SHMT2 and PHGDH protein expression in control and MTHFR-OE cells. MTHFR overexpression suppresses SHMT2 but not PHGDH protein levels. β-actin serves as a loading control. **D** Schematic diagram of the U-^13^C-serine isotope tracing workflow for MTHFR-mediated one-carbon metabolic flux analysis. Quantification of ¹³C incorporation into key downstream one-carbon metabolites (glycine, 5,10-CH₂-THF, SAM, dNTPs) in MTHFR-OE A549 (**E**) and H520 (**F**) cells, detected by U-^13^C-serine isotope tracing assay. **G** Analysis of SHMT2 expression in NSCLC and normal tissues from the TIMER database. **H** Negative correlation between MTHFR and SHMT2 mRNA expression in LUAD samples from the TCGA dataset (Pearson correlation). **I** Cell proliferation was assessed by a CCK-8 assay in cells expressing vector control, MTHFR alone, or MTHFR + SHMT2. **J** Cell cycle distribution analyzed by flow cytometry in the indicated groups. **K** Apoptosis rates measured by flow cytometry in the indicated groups. **L** Immunoblot analysis of pro-survival (p-AKT Ser473, p-ERK1/2, and Bcl-2) and pro-apoptotic (cleaved caspase-3/8) signaling molecules in the rescue experiment. β-actin serves as a loading control. Means ± SEM, *P < 0.05; **P < 0.01; ***P < 0.001; *t*-test.

One-carbon metabolism involves the folate cycle and methionine regeneration pathway (Fig. 5A). Given our metabolomic data indicating that MTHFR overexpression enhances serine metabolic flux (Fig. 3C), we sought to elucidate its mechanistic link to one-carbon metabolism. qPCR analysis of key metabolic genes revealed that MTHFR overexpression significantly downregulated the mRNA expression of SHMT2 and PHGDH (Fig. 5B). Consistent with transcriptional regulation, immunoblotting assays demonstrated that MTHFR overexpression suppressed SHMT2 protein expression, although no significant change in PHGDH protein levels was observed (Fig. 5C). SHMT2 catalyses the conversion of serine to glycine while generating 5,10-methylenetetrahydrofolate (5,10-CH₂-THF), a crucial one-carbon unit donor for nucleotide biosynthesis [15, 16].

To directly delineate the functional impact of MTHFR on one-carbon metabolic flux, we performed U-¹³C-serine isotope tracing assays, with the tracing workflow schematically shown in Fig. 5D. In both A549 and H520 NSCLC cells, MTHFR overexpression significantly reduced the ¹³C incorporation into key downstream one-carbon metabolites, including glycine (M + 2), 5,10-CH₂-THF (M + 2), S-adenosylmethionine (SAM, M + 1), and deoxynucleoside triphosphates (dNTPs, M + 3) (Fig. 5E, F). These data demonstrated that MTHFR suppresses serine-fueled one-carbon metabolic flux in NSCLC cells via transcriptional repression of SHMT2.

We next validated the clinical relevance of SHMT2 in NSCLC. Analysis of the TIMER database revealed that SHMT2 was significantly upregulated in NSCLC tissues compared with adjacent normal lung tissues (Fig. 5G). Further analysis of the TCGA cohort showed that SHMT2 expression was positively correlated with advanced clinical stage in lung adenocarcinoma (LUAD), and high SHMT2 expression was associated with worse overall survival (OS), first progression (FP), and progression-free survival (PFS) in LUAD patients; a consistent trend was observed in lung squamous cell carcinoma (LUSC) but did not reach statistical significance (Supplementary Fig. 5A, B). In line with our in vitro regulatory data, Pearson correlation analysis identified a significant negative correlation between MTHFR and SHMT2 mRNA expression in the TCGA-LUAD cohort (Fig. 5H). To functionally validate the role of SHMT2, we generated SHMT2-knockdown models in A549 and H520 cells (Supplementary Fig. 5C, D). SHMT2 depletion markedly inhibited cell proliferation and increased early apoptosis but did not affect late apoptosis (Supplementary Fig. 5E). Western blot analysis further confirmed that SHMT2 knockdown increased the protein levels of both full-length and cleaved caspase-3 and caspase-8 (Supplementary Fig. 5F). These findings suggest that SHMT2 promotes proliferation and suppresses apoptosis in NSCLC.

To establish the causal role of SHMT2 in MTHFR-mediated tumor suppression, we performed functional rescue experiments by co-overexpressing MTHFR and SHMT2 in NSCLC cells. CCK-8 viability assays showed that concurrent overexpression of SHMT2 significantly reversed the anti-proliferative effect induced by MTHFR overexpression alone (Fig. 5I). Flow cytometry-based cell cycle analysis revealed that SHMT2 co-expression rescued the G2/M phase arrest induced by MTHFR overexpression (Fig. 5J). In parallel, the pro-apoptotic effect of MTHFR overexpression was markedly abrogated by SHMT2 co-overexpression (Fig. 5K). Immunoblotting further confirmed that SHMT2 co-expression reactivated the pro-survival signaling axis, as evidenced by restored phosphorylation of AKT (Ser473) and ERK1/2 (Thr202/Tyr204), upregulated anti-apoptotic protein Bcl-2, and reduced cleavage of pro-apoptotic caspase-3 and caspase-8 in MTHFR-overexpressing cells (Fig. 5L).Collectively, these data demonstrated that MTHFR transcriptionally represses SHMT2 to block serine-fueled one-carbon metabolic flux, thereby inhibiting proliferation and promoting apoptosis in NSCLC.

### Dual control of SHMT2 by SLC25A26: ubiquitination and transcriptional repression

**Fig. 6:**
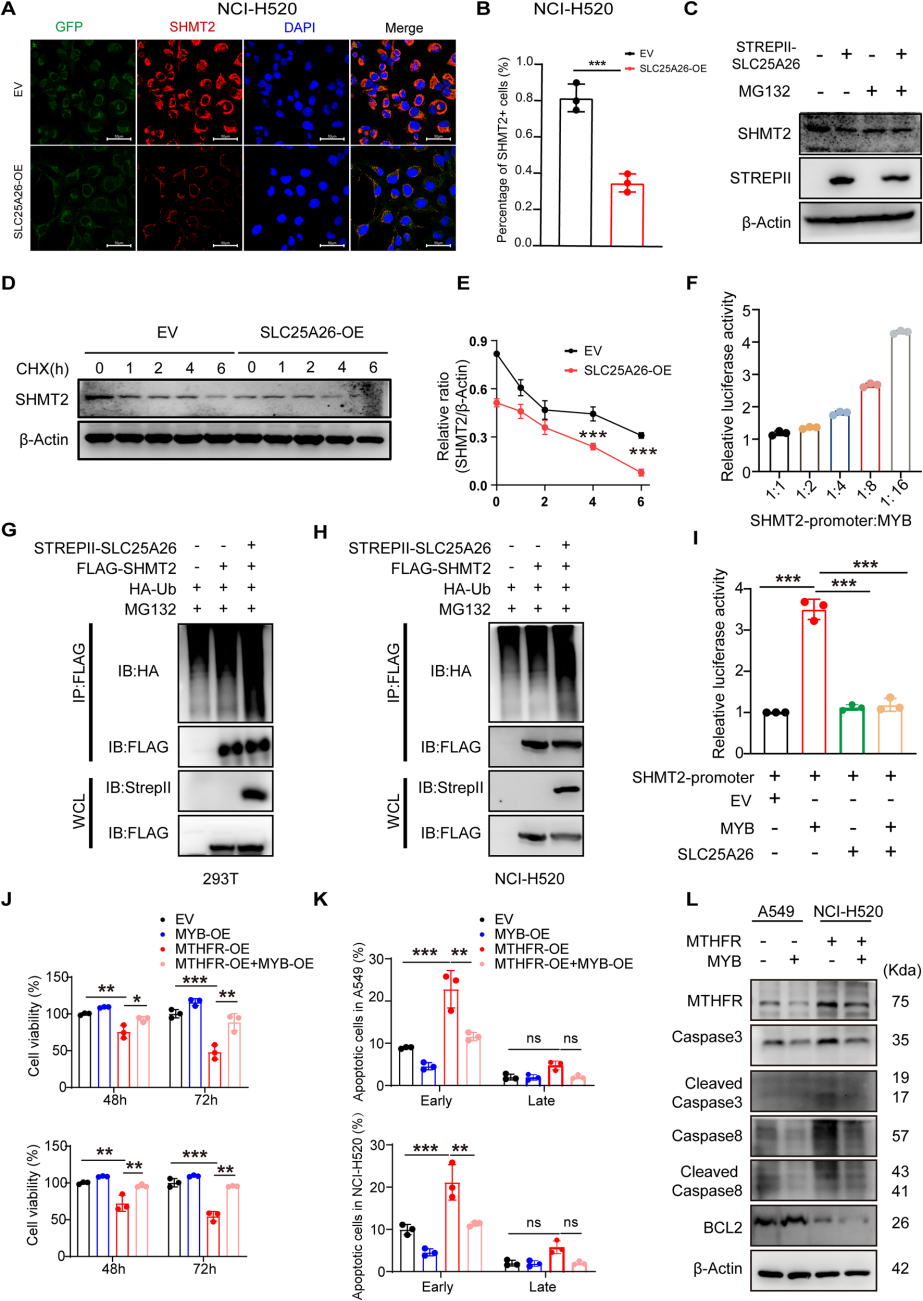
Dual control of SHMT2 by SLC25A26: ubiquitination and transcriptional repression. **A**, **B** Immunofluorescence analysis showing a significant reduction in SHMT2 fluorescence intensity (***P < 0.001) in H520 cells overexpressing SLC25A26 (SLC25A26-OE) compared with that in the empty vector (EV) controls. Scale bars: 50 μm. **C** Restoration of SHMT2 protein levels in SLC25A26-OE cells treated with the proteasome inhibitor MG132 (10 μM, 6 h). **D**, **E** Cycloheximide (CHX, 100 μg/mL) chase assays demonstrating accelerated SHMT2 protein degradation in SLC25A26-OE cells. Half-life quantification is shown (***P < 0.001 vs. EV). **F**, **G** Ubiquitination assays: SHMT2 was immunoprecipitated from cell lysates using anti-ubiquitin (Ub) antibodies, followed by immunoblot analysis with anti-SHMT2. SLC25A26-OE cells exhibited enhanced SHMT2 polyubiquitination. RT‒qPCR analysis of MYB mRNA levels in MTHFR-OE (**H**) and SLC25A26-OE (**I**) cells (*n* = 3 biological replicates). Luciferase reporter assays demonstrating MYB binding to the SHMT2 promoter (**J**) and SLC25A26-mediated suppression of MYB transcriptional activity (**K**). Means ± SEM, *P < 0.05; **P < 0.01; ***P < 0.001; *t*-test.

To elucidate the mechanism underlying the SLC25A26-mediated regulation of SHMT2, we initially conducted immunofluorescence assays and demonstrated that SLC25A26 overexpression markedly reduced SHMT2 protein levels (Fig. 6A, B, Supplementary Fig. 6A, B). This inverse correlation was further validated in clinical datasets (Supplementary Fig. 6C). Proteasome inhibition experiments using MG132 revealed that SLC25A26-driven SHMT2 downregulation was substantially reversed upon proteasomal blockade (Fig. 6C), indicating post-translational regulation via proteasomal degradation. Cycloheximide (CHX) chase assays confirmed accelerated SHMT2 turnover in SLC25A26-overexpressing cells, as the protein half-life was significantly shorter in these cells than in control cells (Fig. 6D, E). Strikingly, ubiquitination assays demonstrated that SLC25A26 overexpression selectively increased SHMT2 ubiquitination, indicating a direct role for SLC25A26 in modulating SHMT2 stability (Fig. 6F, G).

Building on our earlier observation that SLC25A26 regulates AKT phosphorylation (Fig. 3H), we investigated MYB, an AKT-responsive transcription factor linked to SHMT2 expression (Supplementary Fig. 6D). qPCR analysis demonstrated that overexpression of MTHFR and SLC25A26 significantly suppressed MYB mRNA levels in both A549 and H520 cells (Supplementary Fig. 6E, F). Using a luciferase reporter assay, we found that MYB transcriptionally activates the SHMT2 promoter (Fig. 6H). Furthermore, overexpression of SLC25A26 markedly attenuated MYB-dependent transcriptional activation of the SHMT2 promoter (Fig. 6I). These findings led us to hypothesize that MYB may act as a downstream effector of MTHFR. CCK-8 assays revealed that MYB overexpression substantially reversed the antiproliferative effect induced by MTHFR (Fig. 6J). The results of apoptosis assays further confirmed that MYB treatment significantly reversed the pro-apoptotic phenotype caused by MTHFR (Fig. 6K). Finally, western blot analysis confirmed that MYB expression restored the levels of cleaved caspase-3 and caspase-8 and BCL-2 altered by MTHFR (Fig. 6L). Collectively, these data reveal a dual regulatory axis through which SLC25A26 cooperatively suppresses SHMT2 via two distinct mechanisms: post-translational destabilization through ubiquitin‒proteasome-mediated degradation and transcriptional repression via the AKT–MYB signaling pathway.

### In vivo validation of the pathway mechanism mediated by SHMT2 and SLC25A26 in NSCLC

**Fig. 7:**
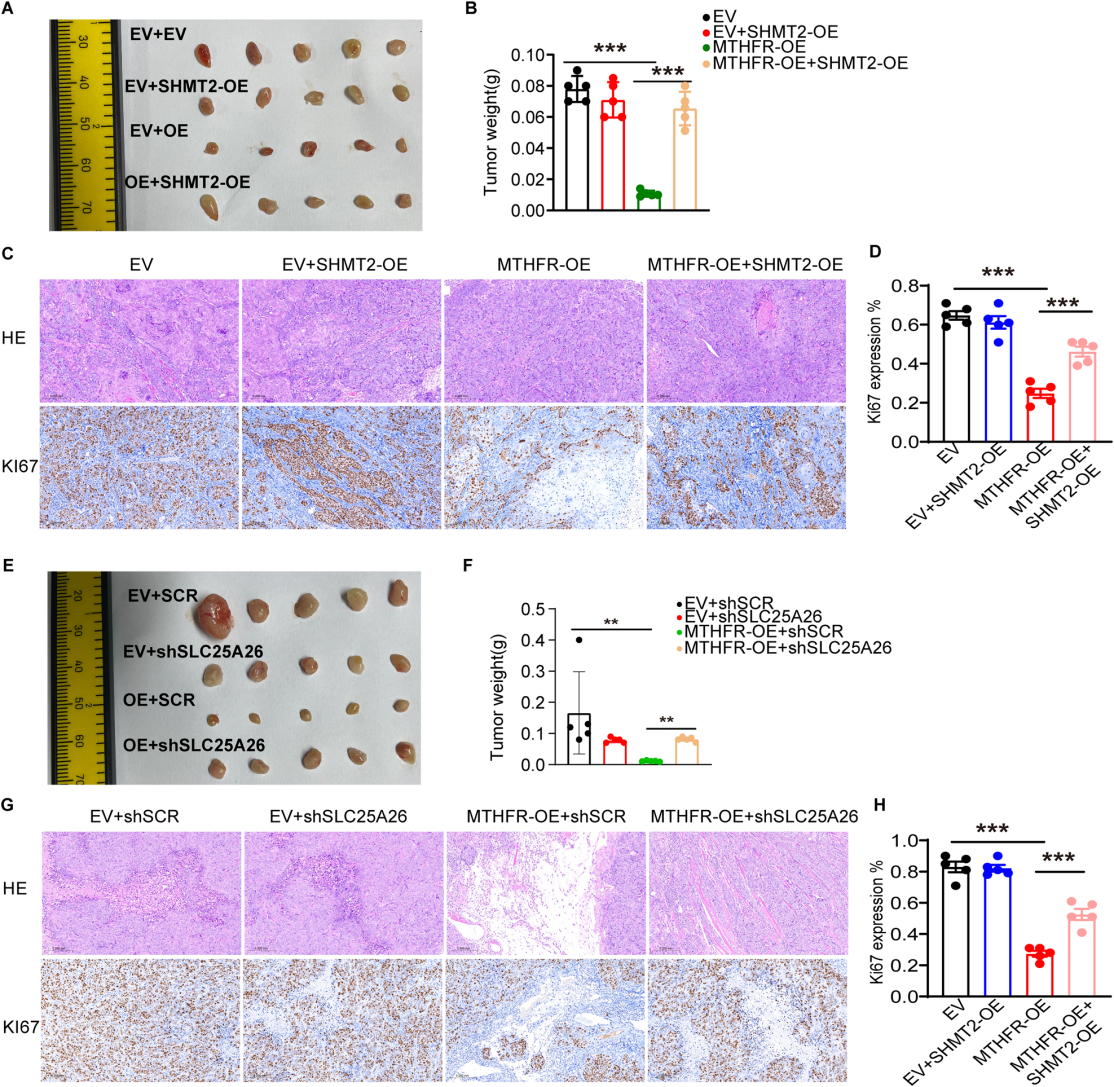
In vivo validation of the MTHFR–SLC25A26–SHMT2 axis in NSCLC progression. SHMT2 co-overexpression rescues MTHFR-mediated tumor suppression: Representative images (**A**) and quantification (**B**) of subcutaneous xenograft weights in nude mice injected with control (EV), MTHFR-overexpressing (MTHFR-OE), or MTHFR-OE + SHMT2-OE H520 cells (*n* = 5 mice/group). **C**, **D** Haematoxylin‒eosin (HE) and immunohistochemical (IHC) staining of tumor tissues showing increased stromal infiltration (black arrows) and increased Ki67-positive proliferating cells (brown nuclei) in MTHFR-OE + SHMT2-OE tumors compared with those in tumors in which MTHFR-OE alone was used. Scale bars: HE = 50 μm; IHC = 100 μm. **E**, **F** Partial restoration of tumor growth in MTHFR-OE xenografts with SLC25A26 knockdown (MTHFR-OE + SLC25A26-KD): Representative images (**E**) and endpoint tumor weights (**F**) (*n* = 5). **G**, **H** HE(G) and IHC(H) staining demonstrating enhanced stromal infiltration and Ki67 positivity in MTHFR-OE + SLC25A26-KD tumors. Means ± SEM, *P < 0.05; **P < 0.01; ***P < 0.001; *t*-test.

Previous in vitro studies demonstrated that SHMT2 and SLC25A26 effectively reversed MTHFR deficiency-induced apoptosis in NSCLC. To further evaluate the functional relevance of these genes in vivo, we established subcutaneous xenograft tumor models in nude mice. Overexpression of SHMT2 significantly attenuated the tumor-suppressive effects of MTHFR, as evidenced by increased tumor weight (Fig. 7A, B) and enhanced tumor infiltration accompanied by increased proportions of KI67-positive proliferating cells, as revealed by haematoxylin‒eosin (HE) staining and immunohistochemical (IHC) analysis (Fig. 7C, D). Strikingly, SLC25A26 knockdown in MTHFR-overexpressing xenografts partially restored the tumor volume to 80% of the control level (Fig. 7E, F) and increased both stromal infiltration and the number of KI67-positive cells (Fig. 7G, H). These collective findings establish that MTHFR regulates NSCLC proliferation and apoptosis through a coordinated mechanism involving SLC25A26-mediated metabolic reprogramming and SHMT2-driven oncogenic rescue.

## Discussion

Our integrated multiomics investigation established MTHFR as a pivotal metabolic regulator in NSCLC, orchestrating tumor suppression through a bidirectional axis involving SLC25A26-mediated mitochondrial SAM transport and SHMT2-dependent one-carbon metabolism. The clinical relevance of this pathway is underscored by the strong correlation between MTHFR/SLC25A26 downregulation and adverse survival outcomes in patients with NSCLC, positioning these metabolic enzymes as potential prognostic biomarkers and therapeutic targets [17–19]. The discovery of the MTHFR–SLC25A26 axis signifies a key advancement in understanding the crosstalk between folate metabolism and mitochondrial SAM homeostasis. Whereas MTHFR is classically recognized for its role in cytosolic folate cycling [20–22], our data reveal its unexpected mitochondrial engagement via direct interaction with SLC25A26, a SAM transporter critical for maintaining mitochondrial methylation capacity [23, 24]. Mechanistically, we further demonstrated that MTHFR enhances SLC25A26 protein stability by inhibiting its ubiquitin-dependent proteasomal degradation, establishing a post-translational regulatory layer that reinforces the MTHFR–SLC25A26 complex. The catalytic dependence of this interaction (abolished in the C677T mutant) suggests that the tumor-suppressive function of MTHFR requires both enzymatic activity and structural competence to recruit SLC25A26. These findings align with emerging evidence that metabolic enzymes often play dual roles as catalytic effectors and scaffolding proteins [25]. The consequent SAM flux modulation provides a mechanistic bridge between folate metabolism and epigenetic regulation, as mitochondrial SAM availability dictates histone/DNA methylation patterns, which impact oncogenic transcription [26–28].

The inhibition of SHMT2 emerges as a pivotal downstream consequence of MTHFR activation, creating a metabolic bottleneck at the serine-to-glycine conversion step. Using U-¹³C-serine isotope tracing, we directly confirmed that MTHFR overexpression suppresses serine-fueled one-carbon metabolic flux, which is attributed to the transcriptional repression of SHMT2. While SHMT2 is widely recognized as a protumorigenic enzyme that fuels nucleotide biosynthesis [8, 29–31], our findings further demonstrate that its genetic ablation induces tumor cell apoptosis, collectively underscoring its indispensable role in sustaining tumor cell survival through metabolic reprogramming [32]. The discovery of SLC25A26-mediated dual regulation of SHMT2—coupling MYB-dependent transcriptional repression with ubiquitin–proteasomal degradation—reveals an unprecedented feed-forward mechanism to constrain one-carbon flux, thereby rewiring metabolic adaptability under stress conditions [33–35]. The clinical implications of this metabolic axis are multifaceted. First, the MTHFR–SLC25A26 interaction defect in the C677T variant may explain the epidemiological associations between this polymorphism and increased cancer risk [36]. Second, the inverse correlation between SHMT2 levels and patient survival suggests its potential as a therapeutic vulnerability—a concept supported by recent efforts to target mitochondrial one-carbon metabolism [5]. Our finding that SHMT2 restoration reverses MTHFR-mediated tumor suppression highlights the need for precise targeting of specific metabolic nodes rather than broad pathway inhibition.

From a translational perspective, the metabolic plasticity observed in our xenograft models has important implications. The partial tumor recovery (80% volume) following SLC25A26 knockdown in MTHFR-overexpressing tumors suggests compensatory activation of alternative survival pathways. This resilience underscores the need for combinatorial targeting strategies, potentially coupling MTHFR activation with inhibitors of parallel metabolic routes such as glutaminolysis [37] or de novo serine synthesis [38]. The cell type-dependent apoptosis patterns (H520 vs. A549) further emphasize the importance of molecular subtyping in therapeutic development, particularly given the distinct metabolic dependencies of LUAD and LUSC subtypes [39].

Several unanswered questions emerge from this work. First, the precise mechanism linking mitochondrial SAM transport (via SLC25A26) to cytosolic AKT signaling remains unclear. Potential mediators include SAM-dependent methylation of phosphatase regulators [40, 41] or mitochondrial retrograde signaling through ROS/mtDNA release [42, 43]. Second, the clinical stage-dependent variation in MTHFR expression suggests dynamic regulation during tumor evolution, possibly involving hypoxic adaptation [44] or therapy-induced metabolic reprogramming [45]. Third, the therapeutic potential of pharmacologically modulating this axis requires exploration.

In conclusion, our study redefines MTHFR as a metabolic linchpin that coordinates mitochondrial transport systems with one-carbon metabolism to constrain NSCLC progression. Our results clarify that MTHFR acts at both post-translational and transcriptional levels: stabilizing SLC25A26 via suppressing ubiquitination and blocking one-carbon flux via repressing SHMT2. The mechanistic triad of enzymatic activity-dependent protein interactions, dual-layer gene regulation, and pathway-specific signaling modulation provides a framework for understanding metabolic plasticity in cancer. These findings suggest that the MTHFR–SLC25A26–SHMT2 axis is a promising target for the development of metabolically informed therapeutic strategies for NSCLC.

## Supplementary information

Supplementary Materials

Original Uncropped Western Blot Images

## Data Availability

The data that support the findings of this study will be made available from the corresponding author upon reasonable request.

## References

[1] Sung H, Ferlay J, Siegel RL, Laversanne M, Soerjomataram I, Jemal A, et al. Global cancer statistics 2020: GLOBOCAN estimates of incidence and mortality worldwide for 36 cancers in 185 countries. CA Cancer J Clin. 2021;71:209–49. 33538338 10.3322/caac.21660

[2] Finley LWS. What is cancer metabolism?. Cell. 2023;186:1670–88. 36858045 10.1016/j.cell.2023.01.038 PMC10106389

[3] Qiu Y, Xie E, Xu H, Cheng H, Li G. One-carbon metabolism shapes T cell immunity in cancer. Trends Endocrinol Metab. 2024;35:967–80. 38925992 10.1016/j.tem.2024.05.010

[4] Martínez-Reyes I, Chandel NS. Mitochondrial one-carbon metabolism maintains redox balance during hypoxia. Cancer Discov. 2014;4:1371–3. 25477105 10.1158/2159-8290.CD-14-1228 PMC4258875

[5] Locasale JW. Serine, glycine and one-carbon units: cancer metabolism in full circle. Nat Rev Cancer. 2013;13:572–83. 23822983 10.1038/nrc3557 PMC3806315

[6] He L, Endress J, Cho S, Wang Y, Liu X, Zhang Y, et al. Suppression of nuclear GSK3 signaling promotes serine/one-carbon metabolism and confers metabolic vulnerability in lung cancer cells. Sci Adv. 2022;8:eabm8786. 35594343 10.1126/sciadv.abm8786 PMC9122323

[7] Tajan M, Hennequart M, Cheung EC, Zani F, Hock AK, Legrave NM, et al. Serine synthesis pathway inhibition cooperates with dietary serine and glycine limitation for cancer therapy. Nat Commun. 2021;12:366. 33446657 10.1038/s41467-020-20223-y PMC7809039

[8] Kim D, Fiske BP, Birsoy K, Freinkman E, Kami K, Possemato R, et al. SHMT2 drives glioma cell survival in ischaemia but imposes a dependence on glycine clearance. Nature. 2015;520:363–7. 25855294 10.1038/nature14363 PMC4533874

[9] Ducker GS, Rabinowitz JD. One-carbon metabolism in health and disease. Cell Metab. 2017;25:27–42. 27641100 10.1016/j.cmet.2016.08.009 PMC5353360

[10] Skibola CF, Smith MT, Kane E, Roman E, Rollinson S, Cartwright RA, et al. Polymorphisms in the methylenetetrahydrofolate reductase gene are associated with susceptibility to acute leukemia in adults. Proc Natl Acad Sci USA. 1999;96:12810–5. 10536004 10.1073/pnas.96.22.12810 PMC23109

[11] Shi Y, Zhang Z, Wang B, Li J, Qin X, Huo Y, et al. Effect of plateletcrit and methylenetetrahydrofolate reductase (MTHFR) C677T genotypes on folic acid efficacy in stroke prevention. Signal Transduct Target Ther. 2024;9:110. 38724491 10.1038/s41392-024-01817-0 PMC11082186

[12] Tibbetts AS, Appling DR. Compartmentalization of mammalian folate-mediated one-carbon metabolism. Annu Rev Nutr. 2010;30:57–81. 20645850 10.1146/annurev.nutr.012809.104810

[13] Manning BD, Toker A. AKT/PKB signaling: navigating the network. Cell. 2017;169:381–405. 28431241 10.1016/j.cell.2017.04.001 PMC5546324

[14] Koh CM, Bezzi M, Low DH, Ang WX, Teo SX, Gay FP, et al. MYC regulates the core pre-mRNA splicing machinery as an essential step in lymphomagenesis. Nature. 2015;523:96–100. 25970242 10.1038/nature14351

[15] Annibal A, Tharyan RG, Schonewolff MF, Tam H, Latza C, Auler MMK, et al. Regulation of the one carbon folate cycle as a shared metabolic signature of longevity. Nat Commun. 2021;12:5025. 34108489 10.1038/s41467-021-23856-9 PMC8190293

[16] Han T, Wang Y, Cheng M, Li Y, Zhang Y, Wang Y, et al. Phosphorylated SHMT2 regulates oncogenesis through m6A modification in lung adenocarcinoma. Adv Sci. 2024;11:e2307834. 10.1002/advs.202307834 PMC11095143 38460155

[17] Han M, Bushong EA, Segawa M, Zhao L, Boassa D, Chaudhari SN, et al. Spatial mapping of mitochondrial networks and bioenergetics in lung cancer. Nature. 2023;615:712–9. 36922590 10.1038/s41586-023-05793-3 PMC10033418

[18] Herrera-Juárez M, Serrano-Gómez C, Bote-de-Cabo H, Paz-Ares L. Targeted therapy for lung cancer: beyond EGFR and ALK. Cancer. 2023;129:1803–20. 37073562 10.1002/cncr.34757

[19] Lahiri A, Maji A, Potdar PD, Saha S, Ghosh SK, Ratha J. Lung cancer immunotherapy: progress, pitfalls, and promises. Mol Cancer. 2023;22:40. 36810079 10.1186/s12943-023-01740-y PMC9942077

[20] Lee Y, Vousden KH, Hennequart M. Cycling back to folate metabolism in cancer. Nat Cancer. 2024;5:701–15. 38698089 10.1038/s43018-024-00739-8 PMC7616045

[21] Blomgren LKM, Huber M, Mackinnon SR, Moffat KA, Jones P, Lemak A, et al. Dynamic inter-domain transformations mediate the allosteric regulation of human 5,10-methylenetetrahydrofolate reductase. Nat Commun. 2024;15:3248. 38622112 10.1038/s41467-024-47174-y PMC11018872

[22] Zarou MM, Rattigan KM, Sarnello D, Li H, Hwang I, Sanchez DJ, et al. Inhibition of mitochondrial folate metabolism drives differentiation through mTORC1 mediated purine sensing. Nat Commun. 2024;15:1931. 38431691 10.1038/s41467-024-46114-0 PMC10908830

[23] Menga A, Palmieri EM, Cianciulli A, Iacobazzi V, Infantino V. SLC25A26 overexpression impairs cell function via mtDNA hypermethylation and rewiring of methyl metabolism. FEBS J. 2017;284:967–84. 28118529 10.1111/febs.14028

[24] Palmieri F. The mitochondrial transporter family SLC25: identification, properties and physiopathology. Mol Aspects Med. 2013;34:465–84. 23266187 10.1016/j.mam.2012.05.005

[25] Guo D, Meng Y, Zhao G, Li X, Wang L, Zhang Y, et al. Moonlighting functions of glucose metabolic enzymes and metabolites in cancer. Nat Rev Cancer. 2025;25:426–46. 40175621 10.1038/s41568-025-00800-3

[26] Mentch SJ, Locasale JW. One-carbon metabolism and epigenetics: understanding the specificity. Ann N Y Acad Sci. 2016;1363:91–98. 26647078 10.1111/nyas.12956 PMC4801744

[27] Bian Y, Li W, Kremer DM, Sajjakulnukit P, Li S, Crespo J, et al. Cancer SLC43A2 alters T cell methionine metabolism and histone methylation. Nature. 2020;585:277–82. 32879489 10.1038/s41586-020-2682-1 PMC7486248

[28] Koenig MJ, Agana BA, Kaufman JM, Kollipara RK, Hight SK, Yan L, et al. STK11/LKB1 loss of function is associated with global DNA hypomethylation and S-adenosyl-methionine depletion in human lung adenocarcinoma. Cancer Res. 2021;81:4194–204. 34045189 10.1158/0008-5472.CAN-20-3199 PMC8373682

[29] Pranzini E, Pardella E, Muccillo L, Nannini M, La Ferla M, Parri M, et al. SHMT2-mediated mitochondrial serine metabolism drives 5-FU resistance by fueling nucleotide biosynthesis. Cell Rep. 2022;40:111233. 35977477 10.1016/j.celrep.2022.111233

[30] Yang C, Zhang J, Liao M. Zhou J. Folate-mediated one-carbon metabolism: a targeting strategy in cancer therapy. Drug Discov Today. 2021;26:817–25. 33316375 10.1016/j.drudis.2020.12.006

[31] McBride MJ, Hunter CJ, Zhang Z, Ryu J, Wang Y, Liu X, et al. Glycine homeostasis requires reverse SHMT flux. Cell Metab. 2024;36:103–.e4. 38171330 10.1016/j.cmet.2023.12.001 PMC11892390

[32] Yang X, Wang Z, Li X, Zhang Y, Liu X, Liu J, et al. SHMT2 desuccinylation by SIRT5 drives cancer cell proliferation. Cancer Res. 2018;78:372–86. 29180469 10.1158/0008-5472.CAN-17-1912

[33] Wu B, Song M, Dong Q, Zhang Y, Li Y, Wang X, et al. UBR5 promotes tumor immune evasion through enhancing IFN-γ-induced PDL1 transcription in triple negative breast cancer. Theranostics. 2022;12:5086–102. 35836797 10.7150/thno.74989 PMC9274738

[34] Birsoy K, Wang T, Chen WW, Freinkman E, Abu-Remaileh M, Sabatini DM. An essential role of the mitochondrial electron transport chain in cell proliferation is to enable aspartate synthesis. Cell. 2015;162:540–51. 26232224 10.1016/j.cell.2015.07.016 PMC4522279

[35] Martínez-Reyes I, Cardona LR, Kong H, Vasan K, McElroy GS, Werner M, et al. Mitochondrial ubiquinol oxidation is necessary for tumour growth. Nature. 2020;585:288–92. 32641834 10.1038/s41586-020-2475-6 PMC7486261

[36] Tiseo M, Giovannetti E, Tibaldi C, Boni L, Capelletti M, Loi M, et al. Pharmacogenetic study of patients with advanced non-small cell lung cancer (NSCLC) treated with second-line pemetrexed or pemetrexed-carboplatin. Lung Cancer. 2012;78:92–99. 22889494 10.1016/j.lungcan.2012.07.009

[37] Altman BJ, Stine ZE, Dang CV. From Krebs to clinic: glutamine metabolism to cancer therapy. Nat Rev Cancer. 2016;16:749. 28704361 10.1038/nrc.2016.114

[38] Maddocks ODK, Berkers CR, Mason SM, Zheng L, Blyth K, Gottlieb E, et al. Serine starvation induces stress and p53-dependent metabolic remodelling in cancer cells. Nature. 2013;493:542–6. 23242140 10.1038/nature11743 PMC6485472

[39] Dejure FR, Eilers M. MYC and tumor metabolism: chicken and egg. EMBO J. 2017;36:3409–20. 29127156 10.15252/embj.201796438 PMC5709748

[40] Liu PS, Wang H, Li X, Karwacz K, Xiao M, Lu Z, et al. α-ketoglutarate orchestrates macrophage activation through metabolic and epigenetic reprogramming. Nat Immunol. 2017;18:985–94. 28714978 10.1038/ni.3796

[41] Sun M, Zhao M, Li R, Zhang Y, Wang Y, Zhang Y, et al. SHMT2 promotes papillary thyroid cancer metastasis through epigenetic activation of AKT signaling. Cell Death Dis. 2024;15:87. 38272883 10.1038/s41419-024-06476-1 PMC10811326

[42] Qiu S, Zhong X, Meng X, Li Y, Zhang Y, Liu X, et al. Mitochondria-localized cGAS suppresses ferroptosis to promote cancer progression. Cell Res. 2023;33:299–311. 36864172 10.1038/s41422-023-00788-1 PMC10066369

[43] Cheung EC, Vousden KH. The role of ROS in tumour development and progression. Nat Rev Cancer. 2022;22:280–97. 35102280 10.1038/s41568-021-00435-0

[44] Samanta D, Semenza GL. Metabolic adaptation of cancer and immune cells mediated by hypoxia-inducible factors. Biochim Biophys Acta Rev Cancer. 2018;1870:15–22. 30006019 10.1016/j.bbcan.2018.07.002

[45] Hu J, Wang SG, Hou Y, Liu J, Wang Y, Li X, et al. Multi-omic profiling of clear cell renal cell carcinoma identifies metabolic reprogramming associated with disease progression. Nat Genet. 2024;56:442–57. 38361033 10.1038/s41588-024-01662-5 PMC10937392

