# Supplementary material for: MTHFR functions as a metabolic checkpoint in NSCLC through SLC25A26-mediated SHMT2 inhibition
Source: Cell Death Dis. 2026 Jun 1;17(1):680. doi: 10.1038/s41419-026-08874-z (PMC13434245; doi:10.1038/s41419-026-08874-z)
Supplement: Supplementary file 1 [file 41419_2026_8874_MOESM1_ESM.pdf]

Supplementary Materials for

MTHFR Functions as a Metabolic Checkpoint in NSCLC through SLC25A26-Mediated SHMT2 Inhibition

Correspondence to: Lan li[1,2 * #], YiRan Yang[1,2 #], ShiQing Wang[1 #], Dan Li[1,2], ChuMao Chen[3], XinMei Mu[3], JiaXin Wu[3], Jin Yuan[3 *]

1. Shanghai Chest Hospital, Shanghai Jiao Tong University School of Medicine
2. Shanghai Key Laboratory of Thoracic Tumor Biotherapy
3. Shanghai Jiao Tong University School of Medicine

This word file includes:

Figures. S1 to S6
Table S1 to S2

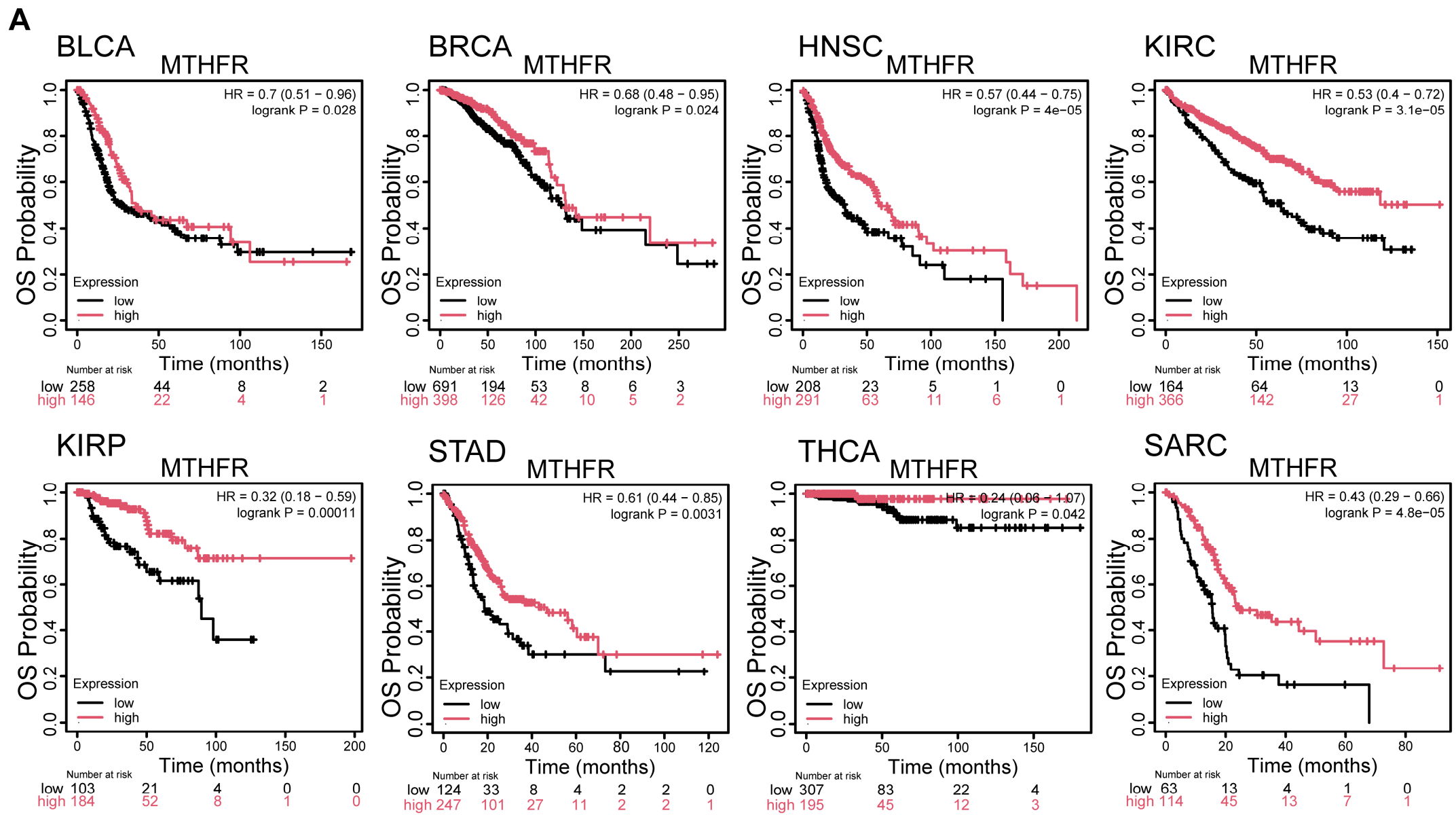

Figure S1 Survival analysis of MTHFR across various cancer types.
Prognostic significance of MTHFR expression in bladder urothelial carcinoma (BLCA), breast cancer (BRCA), head and neck squamous cell carcinoma (HNSC), kidney renal clear cell carcinoma (KIRC), kidney renal papillary cell carcinoma (KIRP), stomach adenocarcinoma (STAD), thyroid carcinoma (THCA), and sarcoma (SARC).

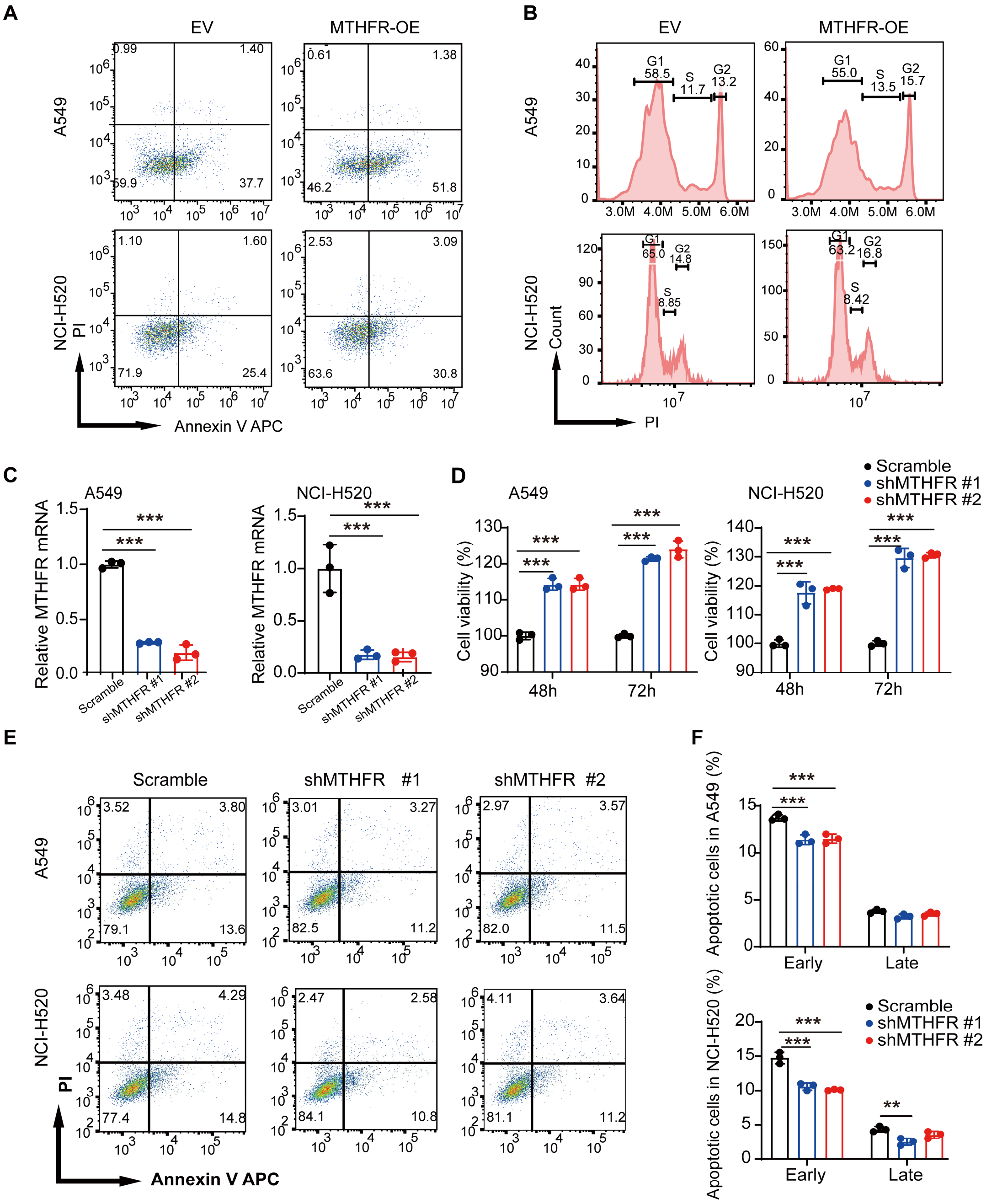

Figure S2 Dual role of MTHFR in proliferation and apoptosis modulation.
(A) MTHFR overexpression triggers apoptosis in NSCLC cells. Flow cytometry plots of Annexin V-APC/PI costaining of the targeting plasmid (MTHFR-OE). **(B)** MTHFR overexpression induces cell cycle arrest. DNA content histograms from propidium iodide (PI) staining showing cell cycle phase redistribution (G1, S, and G2) in MTHFR-OE cells versus EV controls. Percentages indicate population proportions (mean±SD, n=3). **(C)** Validation of efficient MTHFR knockdown. qPCR analysis demonstrating mRNA-level knockdown efficiency in A549 and NCI-H520 cells transfected with two independent shMTHFR constructs (#1, #2) versus the scramble control. **(D)** MTHFR knockdown enhances NSCLC cell proliferation. CCK-8 assay revealing the pro-proliferative effects of shMTHFR transfection at 48 h and 72 h post-treatment. **(E–F)** MTHFR knockdown

confers resistance to apoptosis. E) Representative flow cytometry dot plots of Annexin V/PI staining. F) Statistics of early and late apoptosis in shMTHFR-transfected cells. Means ± SEM, *$P < 0.05$; **$P < 0.01$; ***$P < 0.001$; *t*-test.

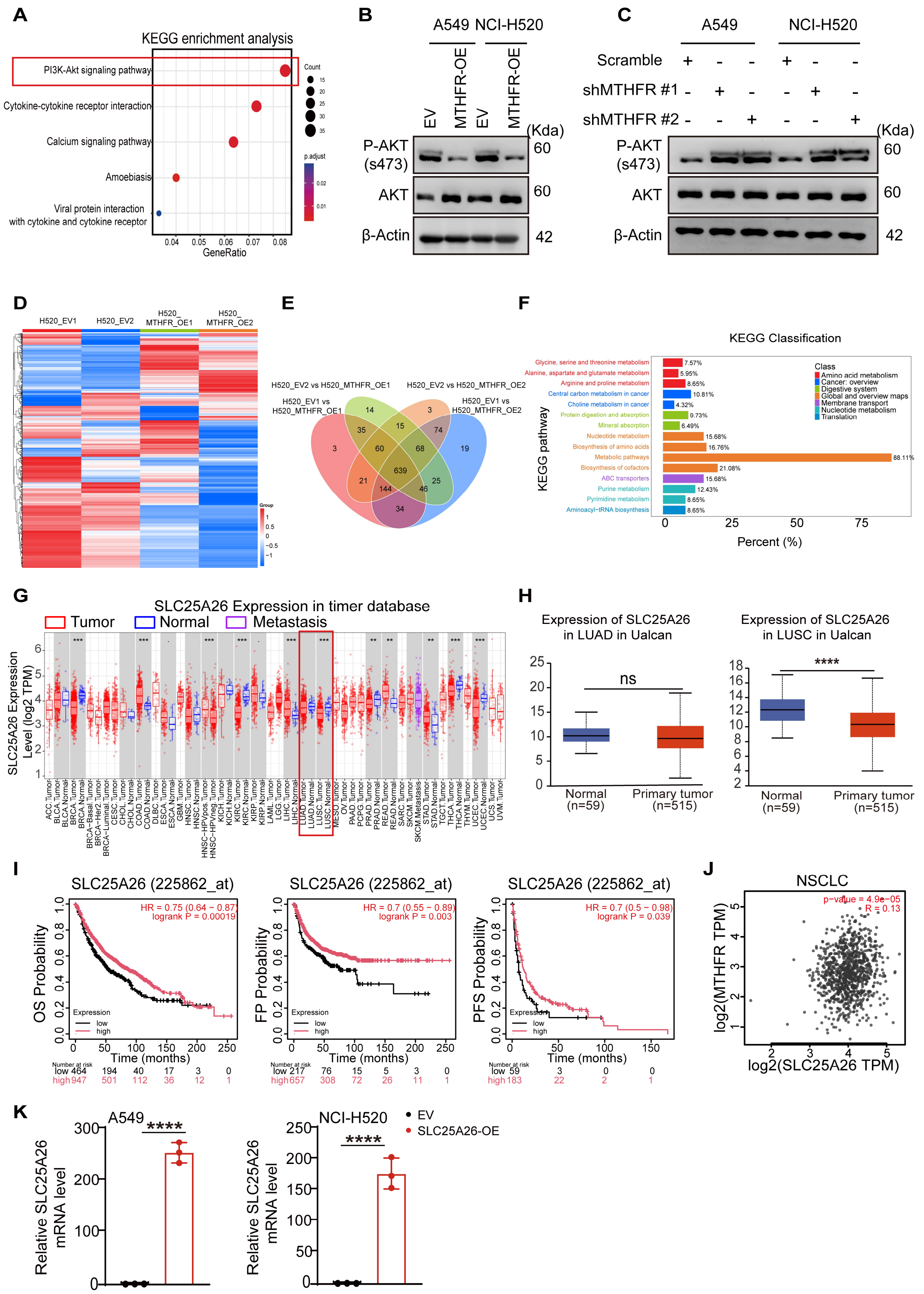

Figure S3 Mechanistic linkage between MTHFR-mediated PI3K-AKT signalling and the clinical relevance of SLC25A26 in NSCLC.

(A) Overexpression of MTHFR inhibits PI3K-AKT signalling transduction. KEGG pathway enrichment analysis of RNA-seq data from MTHFR-overexpressing NSCLC cells. The top significantly enriched pathways are shown, with PI3K–AKT signalling highlighted (red box). **(B–C)** Bidirectional regulation of AKT phosphorylation by MTHFR manipulation. B)

Western blot analysis of phospho-AKT (Ser473) in A549 and NCI-H520 cells transfected with an empty vector (EV) or a MTHFR overexpression plasmid (MTHFR-OE). C) Corresponding analysis of cells transfected with scramble shRNA or shMTHFR constructs. **(D)** Heatmap of metabolites from untargeted metabolomics analysis comparing the H520-EV and H520-MTHFR-OE groups (n=2 biological replicates). **(E)** Venn diagram analysis of untargeted metabolomics data between the H520-EV1/2 and H520-MTHFR-OE1/2 groups, with 639 metabolites overlapping in the intersection. **(F)** KEGG pathway enrichment analysis based on untargeted metabolomics data from the H520-EV1/2 and H520-MTHFR-OE1/2 groups. **(G–H)** Pancancer and NSCLC-specific expression patterns of SLC25A26. D) SLC25A26 mRNA expression across 33 TCGA cancer types (GTEx normal tissues as reference). E) Differential expression in lung adenocarcinoma (LUAD) versus lung squamous cell carcinoma (LUSC) from the TCGA cohort. **(I)** Prognostic significance of SLC25A26 in NSCLC. Kaplan–Meier survival curves showing overall survival (OS), progression-free survival (PFS), and first progression (FP) in the TCGA-LUAD/LUSC cohort stratified by SLC25A26 expression. **(J)** Analysis of the correlation between MTHFR and SLC25A26 expression in non-small cell lung cancer (NSCLC). **(K)** Validation of SLC25A26 overexpression efficiency. qPCR analysis demonstrating SLC25A26 mRNA levels in A549 and NCI-H520 cells transfected with the SLC25A26-OE plasmid versus those in the EV control cells. Means ± SEM, $^{*}P < 0.05$; $^{**}P < 0.01$; $^{***}P < 0.001$; *t*-test.

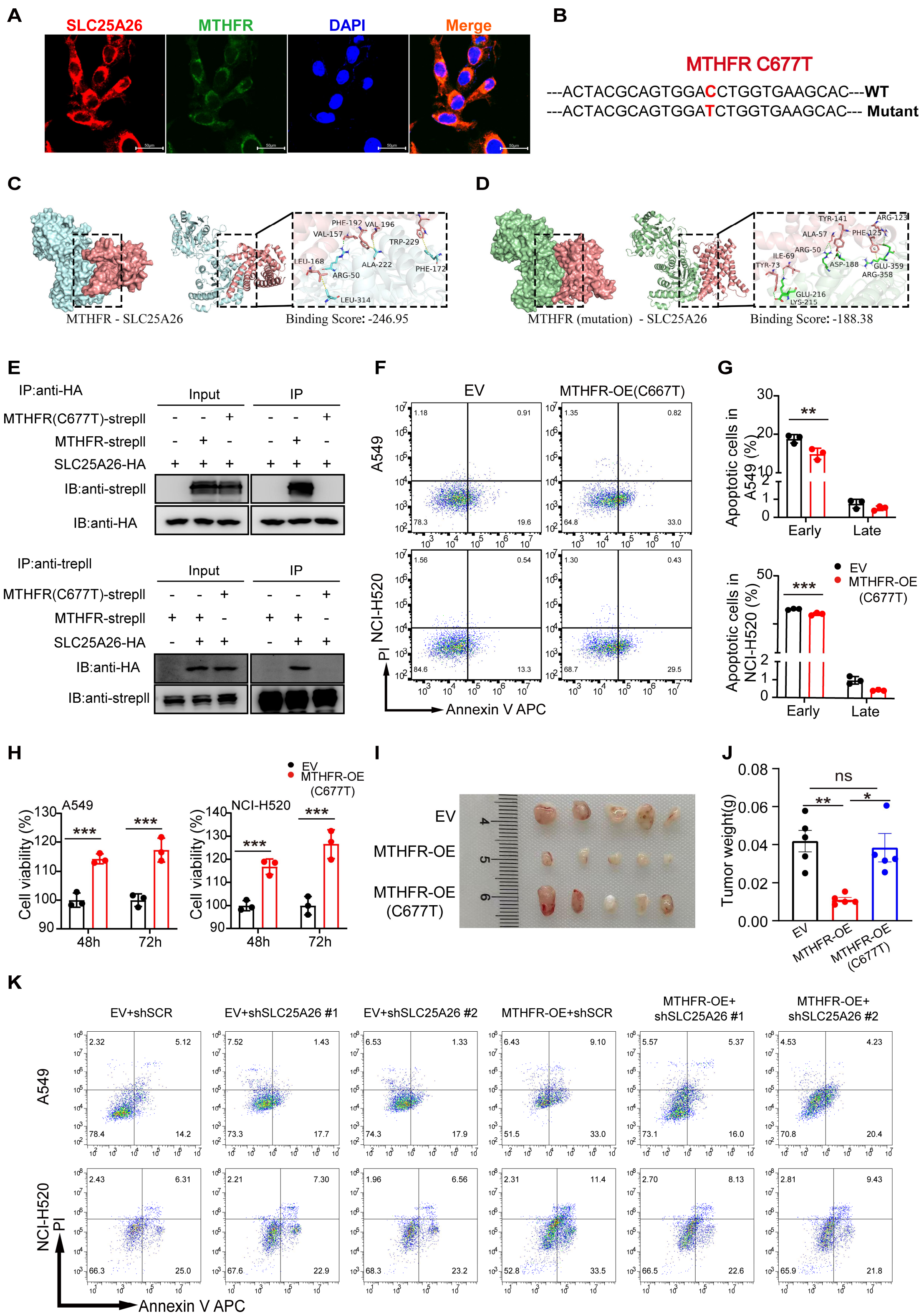

Figure S4 The MTHFR C677T polymorphism abrogates SLC25A26 interaction and abolishes tumor suppression

(A) Confocal immunofluorescence showing colocalization of MTHFR (green) and SLC25A26 (red) in H520 cells. Scale bar = 50 μm. **(B)** Schematic representation of the catalytically inactive MTHFR-C677T (A222V) mutant. **(C-D)** Molecular docking models

showing the binding interface between wild-type MTHFR and SLC25A26 (C) and the disrupted interaction in the MTHFR A222V mutant (D). **(E)** Co-immunoprecipitation showing that the MTHFR-C677T mutant loses physical interaction with SLC25A26. **(F-G)** Flow cytometry analysis showing that MTHFR-C677T overexpression inhibits apoptosis in A549 and H520 cells. Representative dot plots (F) and quantification (G). **(H)** CCK-8 assays showing that MTHFR-C677T promotes proliferation in a time-dependent manner in NSCLC cells. **(I-J)** Subcutaneous xenograft tumor images (I) and growth curve quantification (J) showing that MTHFR overexpression suppresses tumor growth, whereas the C677T mutant abolishes this effect. **(K)** Flow cytometry showing that SLC25A26 knockdown restores apoptosis in MTHFR-overexpressing H520 cells. Means ± SEM, $*P < 0.05$; $**P < 0.01$; $***P < 0.001$; *t*-test.

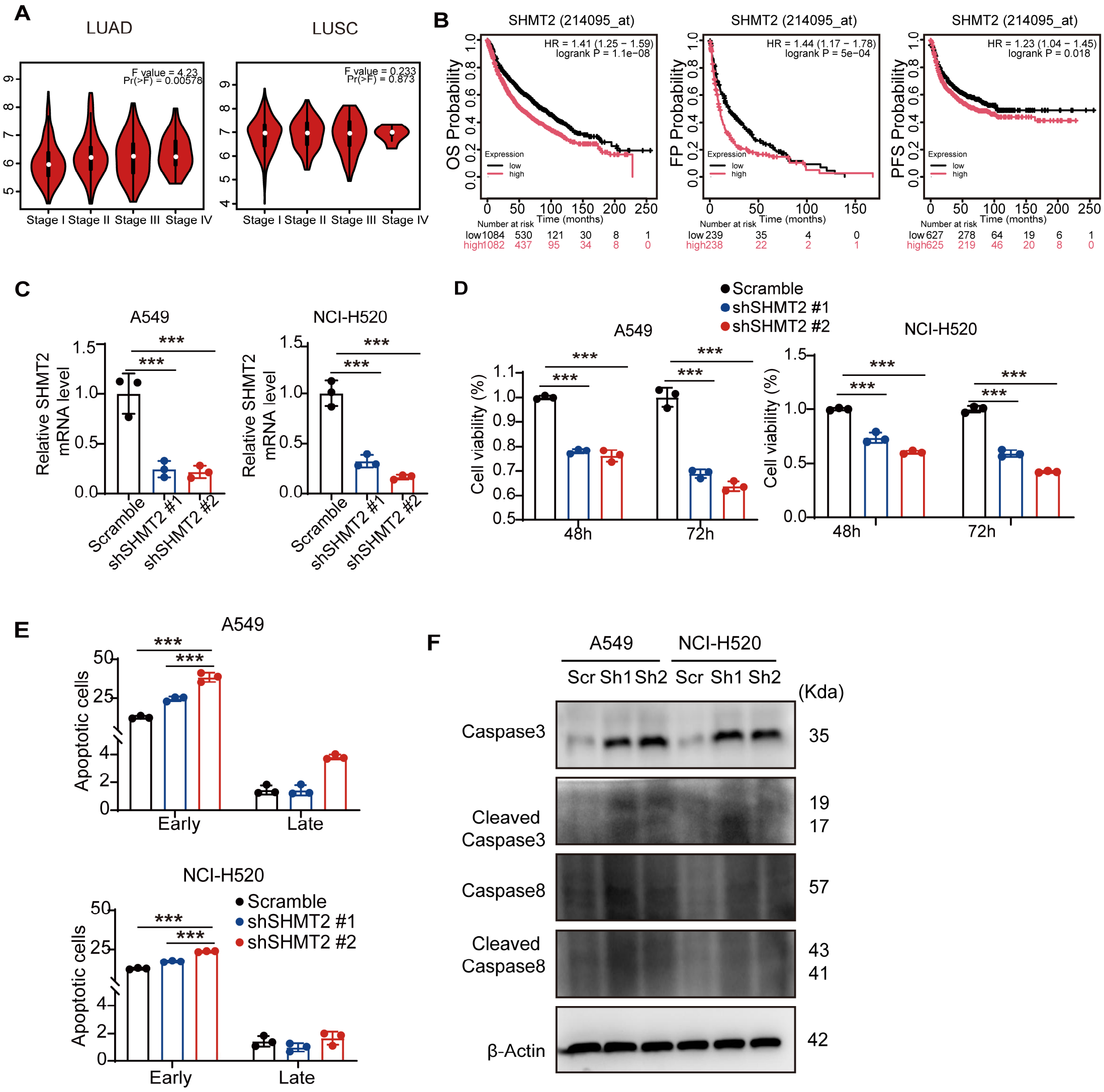

Figure S5 Oncogenic role of SHMT2 in NSCLC.

(A) Stage-dependent SHMT2 expression in NSCLC subtypes. Box plots comparing SHMT2 mRNA levels in LUAD and LUSC across TNM stages (I–IV) from the TCGA cohort. **(B)** Prognostic significance of SHMT2 in NSCLC. Kaplan–Meier survival curves showing overall survival (OS), progression-free survival (PFS), and first progression (FP) in the TCGA-LUAD/LUSC cohorts stratified by SHMT2 expression. **(C)** Validation of SHMT2 knockdown efficiency. qPCR analysis of SHMT2 mRNA in A549 and NCI-H520 cells transfected with shSHMT2#1/#2 versus scramble control. **(D)** SHMT2 knockdown suppresses NSCLC proliferation. CCK-8 viability assay showing time-dependent growth inhibition. **(E)** SHMT2 depletion induces apoptosis in NSCLC cells. Statistics of early and late apoptosis in shSHMT2-transfected cells. **(F)** SHMT2 depletion activates apoptotic signalling. Immunoblot analysis reveals increased levels of both full-length and cleaved forms of caspase-3 and caspase-8 in SHMT2-knockdown A549 and H520 cells compared with control cells. β-Actin serves as a loading control. Means ± SEM, *$P < 0.05$; **$P < 0.01$; ***$P < 0.001$; *t*-test.

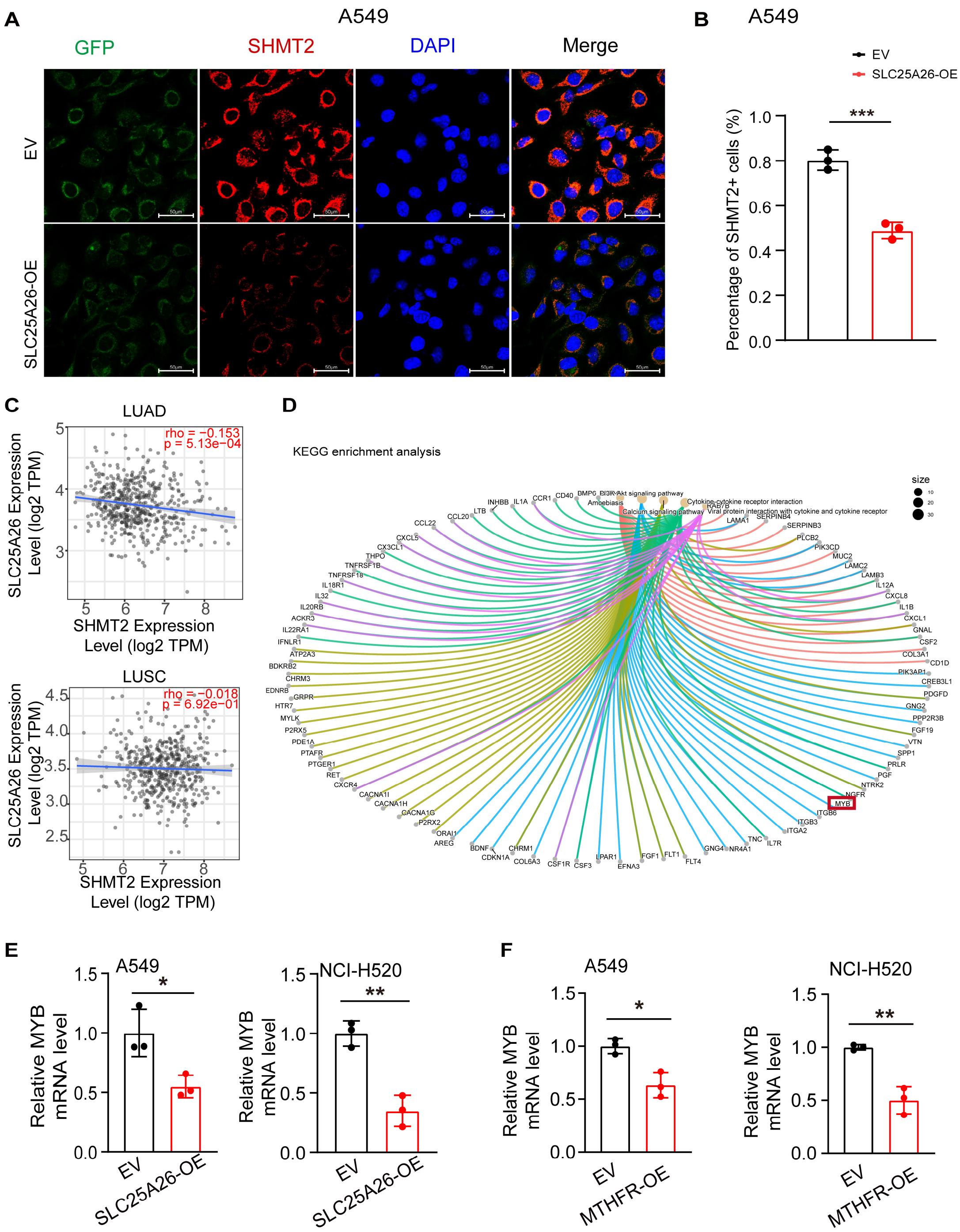

Figure S6 Metabolic crosstalk between SLC25A26 and SHMT2 in NSCLC progression.

(A–B) SLC25A26 overexpression suppresses SHMT2 expression in NSCLC cells. A) Representative immunofluorescence images of SHMT2 (red) and SLC25A26 (green) in A549 cells transfected with empty vector (EV) or SLC25A26-overexpressing plasmid (OE). Nuclei were counterstained with DAPI (blue). Scale bar: 50 μm. B) Quantification of SHMT2 fluorescence intensity normalized to that of DAPI (n = 3 fields per group). **(C)** Inverse correlation between SHMT2 and SLC25A26 in NSCLC subtypes. Scatter plots showing a significant negative correlation between SHMT2 and SLC25A26 mRNA levels in the TCGA-LUAD and TCGA-LUSC cohorts. The expression values are

log2-transformed (TPM). **(D)** KEGG pathway analysis identifies MYB suppression of AKT signalling. RNA-seq profiling of MTHFR-overexpressing NCI-H520 cells reveals significant downregulation of MYB expression in the PI3K-AKT signalling pathway. **(E–F)** Overexpression of MTHFR and SLC25A26 downregulates MYB transcription. qPCR analysis of MYB mRNA levels in A549 and H520 cells following overexpression of MTHFR (E) or SLC25A26 (F). Means ± SEM, $^{*}P < 0.05$; $^{**}P < 0.01$; $^{***}P < 0.001$; *t*-test.

Table S1. Primer pairs used for qRT-PCR

**Name**	**Sequence Forward**	**Sequence Reverse**
MTHFR	AGATGGCTACCACAGAGGTGCT	TGGGACGTACATCTTCCTCTCG
MTHFD1L	CGCACACCTGAATGTCAACTCC	CGTGGATGTCTCCAGTCAAGTG
MTHFD1	GTTGAAGGAGCAAGTACCTGG	GGTAGCTGCACTAAGAACCCA
MTHFD2	CTCCTTGTTCAGTTGCCTCTTCC	CTGATCCAAACACATTCGTCCTAC
SHMT1	TGAACACTGCCATGTGGTGACC	CTCTTTGCCAGTCTTGGGATCC
SHMT2	CCCTTCTGCAACCTCACGAC	TGAGCTTATAGGGCATAGACTCG
DHFR1	CATGGTCTGGATAGTTGGTGGC	GTGTCACTTTCAAAGTCTTGCATG
DHFR2	CAGGTATTTCCAGAGAATGACCAC	CTCAGGAATGGAGAACCAGGTC
SLC25A12	GGGCTTCTTTGGACTCTACAGG	GGAAGTGGAACAGAGCCATCTC
SLC25A13	AGATGGTTCGGTCCCACTTGCA	ACCAGTGGTGATTTCTCCTGCC
SLC25A18	GCAGTGAACCTCACTCTGGTCA	GGCAAGCATCTCCATCTTCAGG
SLC25A22	GTCAACGAGGACACCTACTCTG	GGAAGTAGACCACCTGTGCGAT
SLC25A26	CAGCAGTCTGTGGAGCTTTTGC	TGCAGGACAGAGAGCACATTCC
SLC25A28	AGCCACTGTCACCACGCACATG	GTCAGGCTGTAGACTCTGCATC
SLC25A32	GCGTCTTATCCAACCTTGCGCT	GTTTCCAAATGGTAGTCAAGCAATG
SLC25A48	CACCCTCTGGACACAGTCAAGA	TTGTAGACGGCAATGCTGGCGA
MYB	GGGAACAGATGGGCAGAAATCG	GCTGGCTTTTGAAGACTCCTGC
β-Actin	CACCATTGGCAATGAGCGGTTC	AGGTCTTTGCGGATGTCCACGT

Table S2. Antibodies

Target	Vendor	Cat#	Dilution
MTHFR	ABclonal	A24013	1:1000
β-Actin	ABclonal	AC026	1:5000
Caspase-3	CST	9662	1:1000
Cleaved Caspase-3	CST	9664	1:1000
Caspase-8	CST	9746	1:1000
Cleaved Caspase-8	CST	9496	1:1000
BCL2	CST	15071	1:1000
p-Akt (Ser473)	CST	4060	1:1000
Akt	CST	9272	1:1000
ERK1/2	CST	4695	1:1000
p-ERK1/2	CST	4370	1:1000
SHMT2	ABclonal	A21803	1:1000
PHGDH	ABclonal	A22129	1:1000
HA tag	Proteintech	51064-2-AP	1:1000
StrepII tag	ABclonal	AE066	1:5000
Flag tag	CST	8146	1:1000
