# Supplementary material for: MTHFR functions as a metabolic checkpoint in NSCLC through SLC25A26-mediated SHMT2 inhibition
Source: Cell Death Dis. 2026 Jun 1;17(1):680. doi: 10.1038/s41419-026-08874-z (PMC13434245; doi:10.1038/s41419-026-08874-z)

**Fig. 1C**

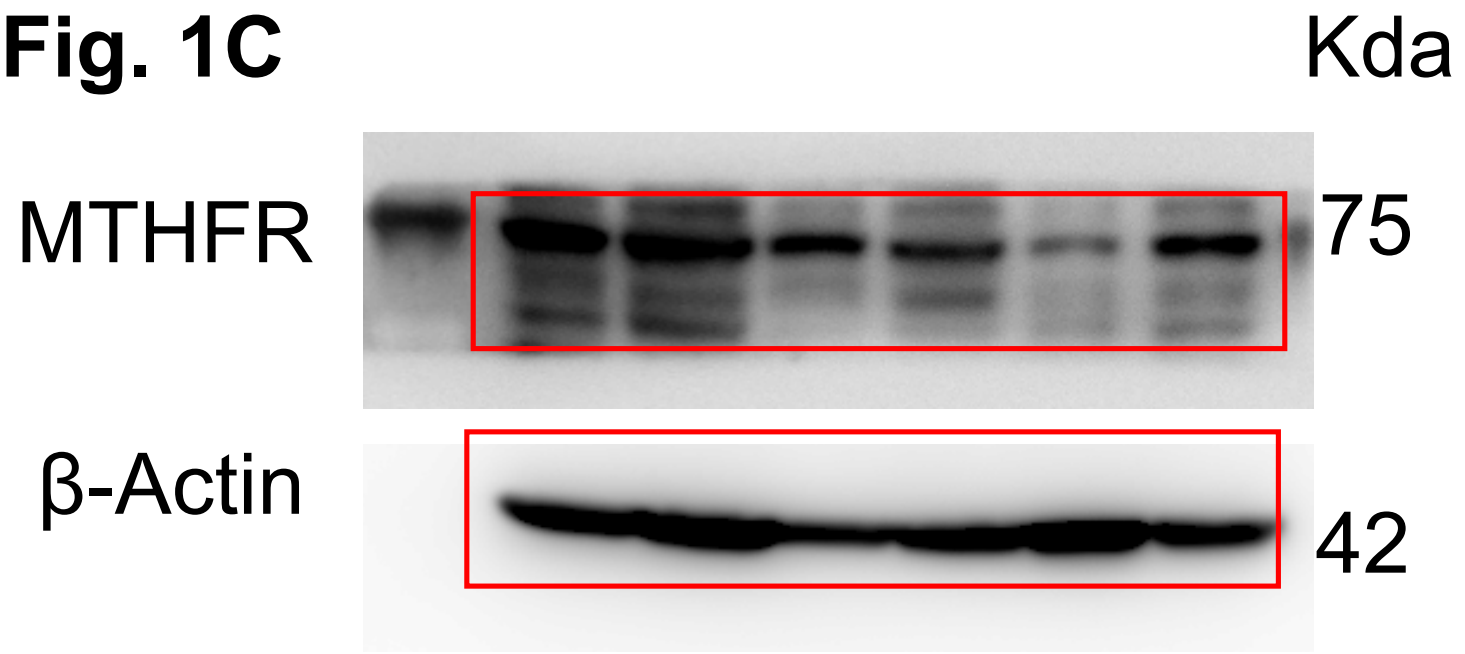
Kda
MTHFR
75
β-Actin
42

**Fig. 2G**

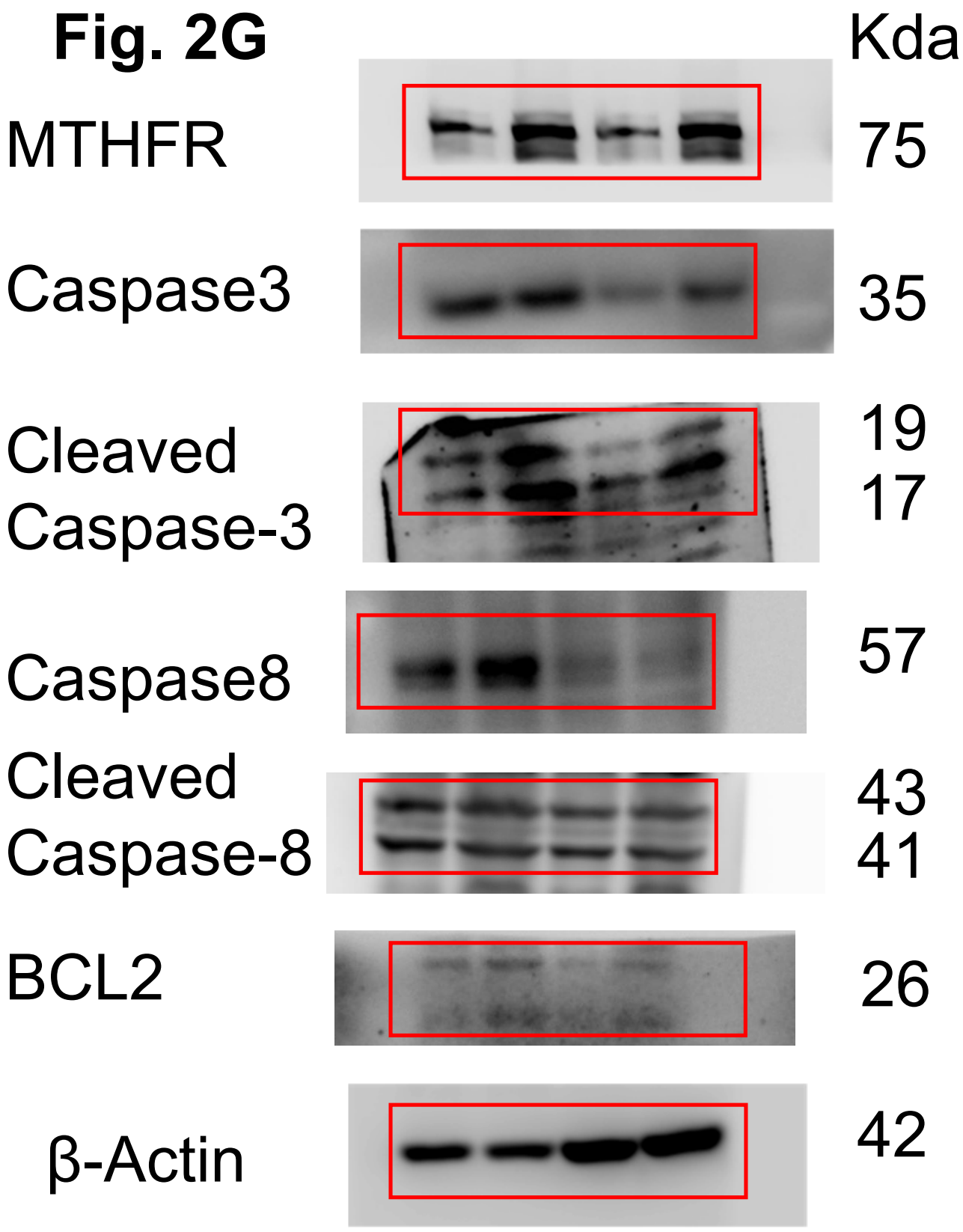
Kda
MTHFR
75
Caspase3
35
Cleaved Caspase-3
19
17
Caspase8
57
Cleaved Caspase-8
43
41
BCL2
26
β-Actin
42

**Fig. 3F**

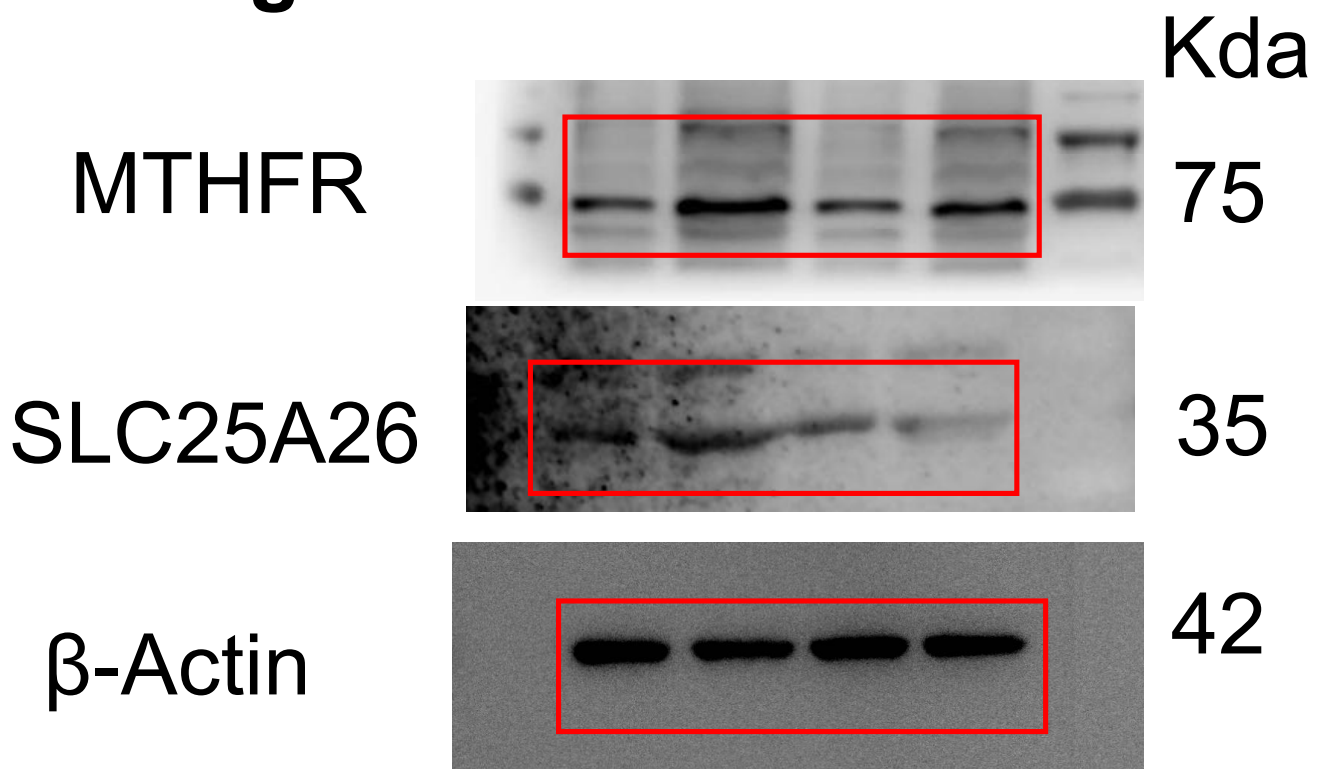


**Fig. 3G**

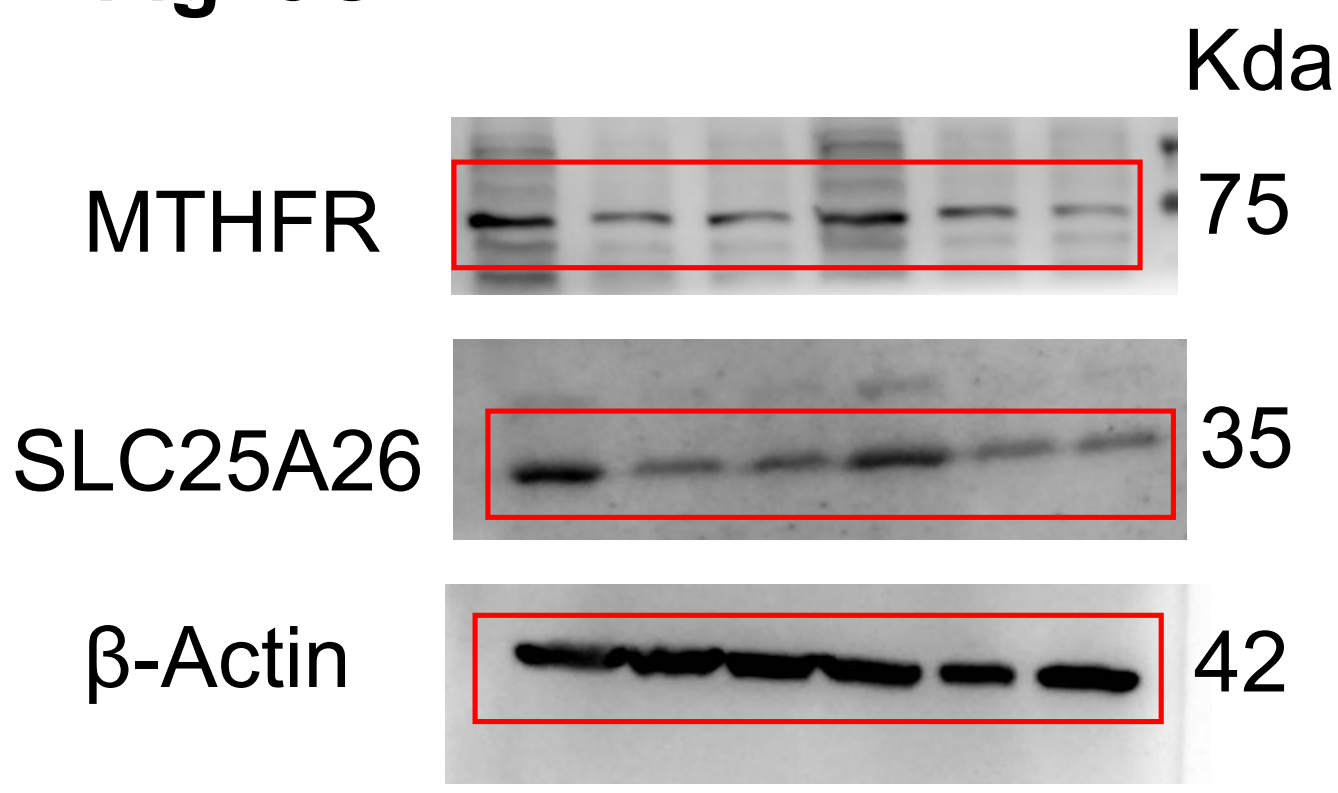


**Fig. 3L**

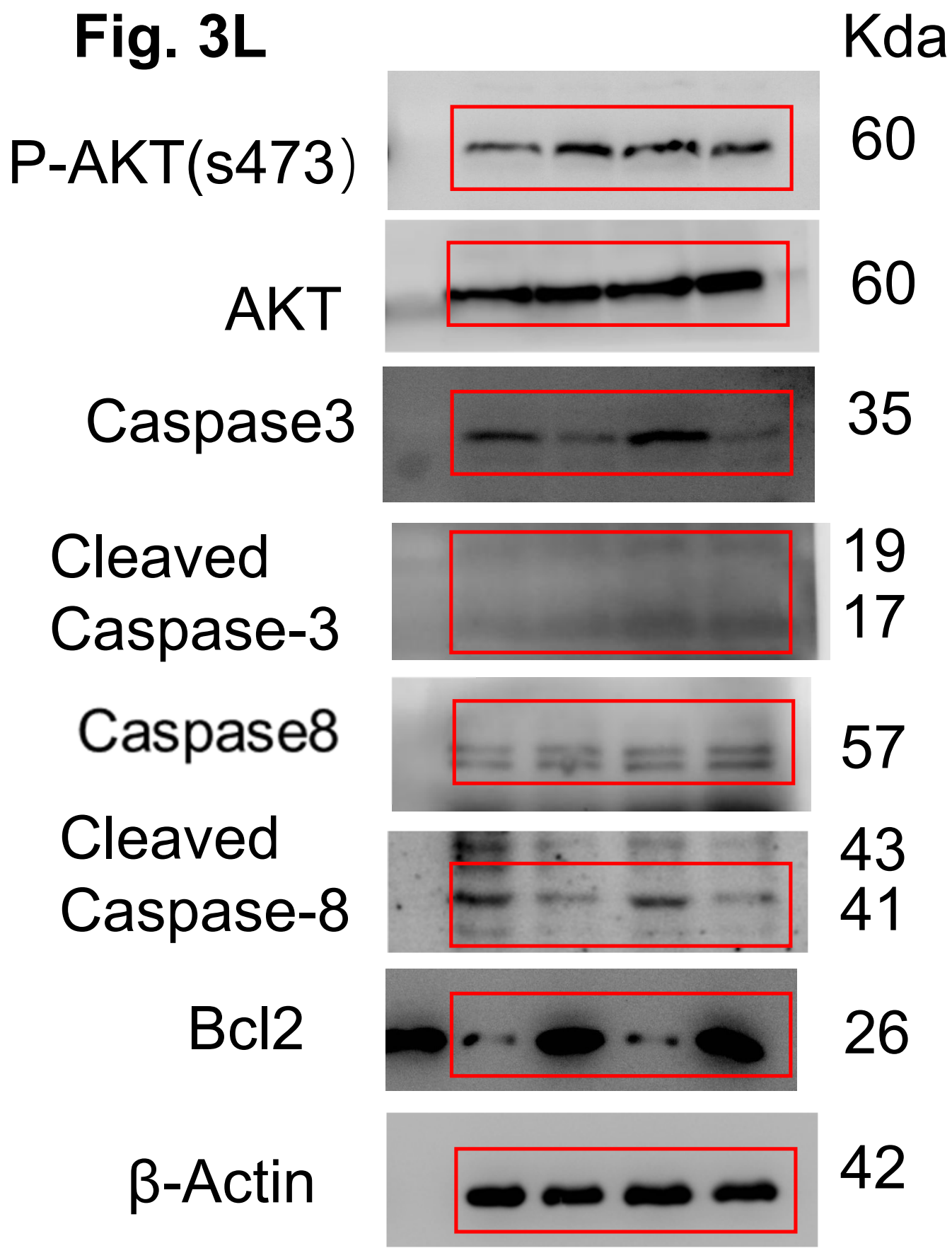


**Fig. 3M**

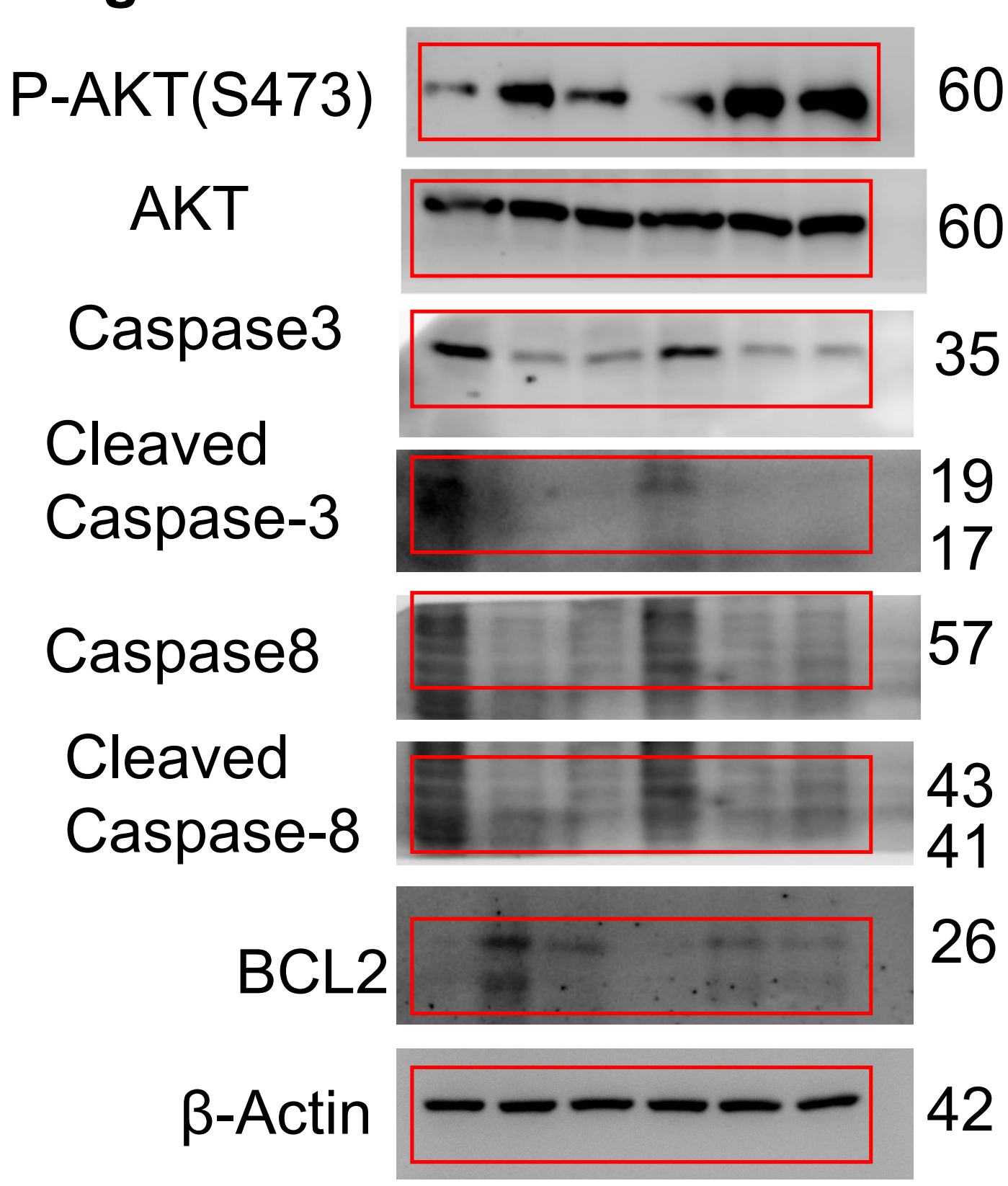

**Fig. 4A**

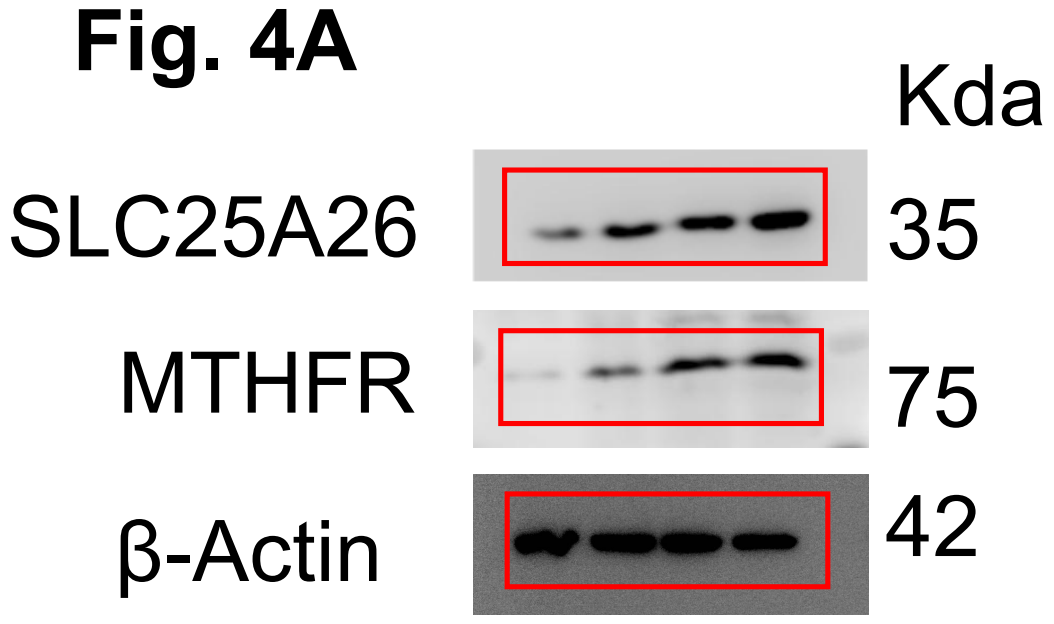


**Fig. 4B**

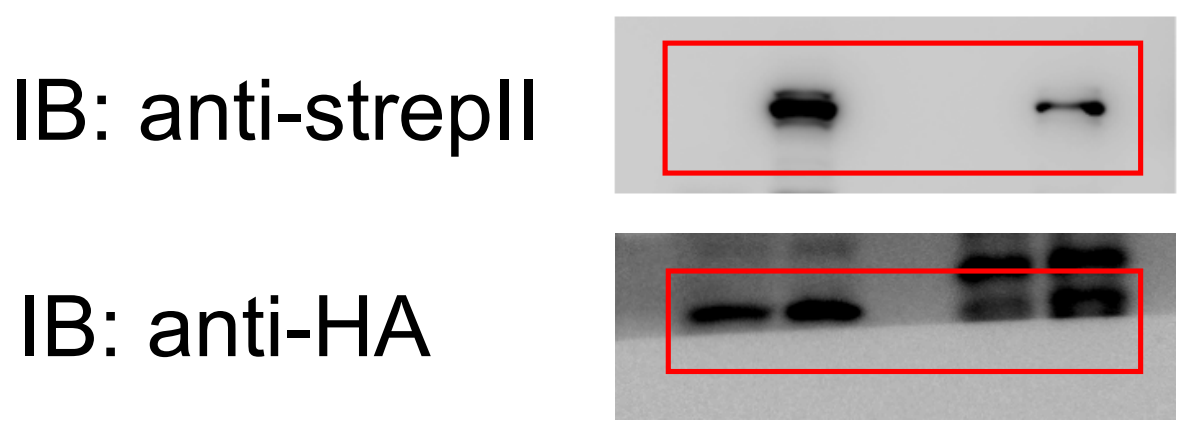


**Fig. 4C**

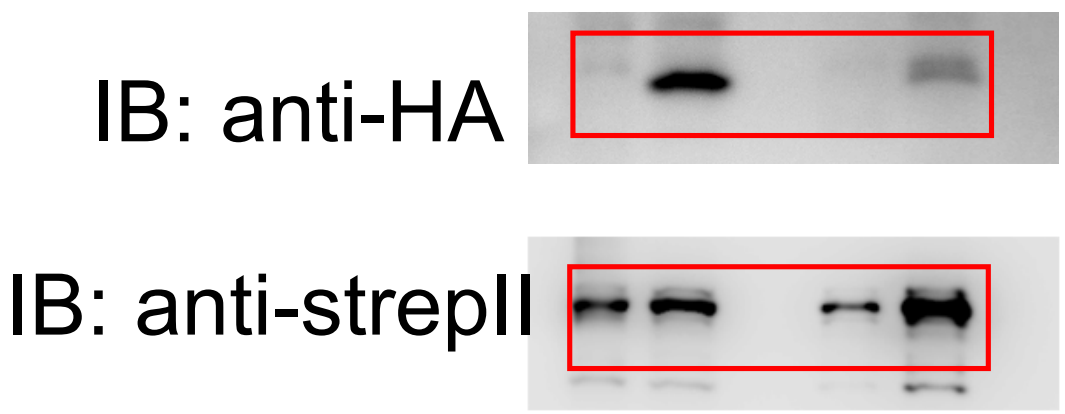


**Fig. 4D**

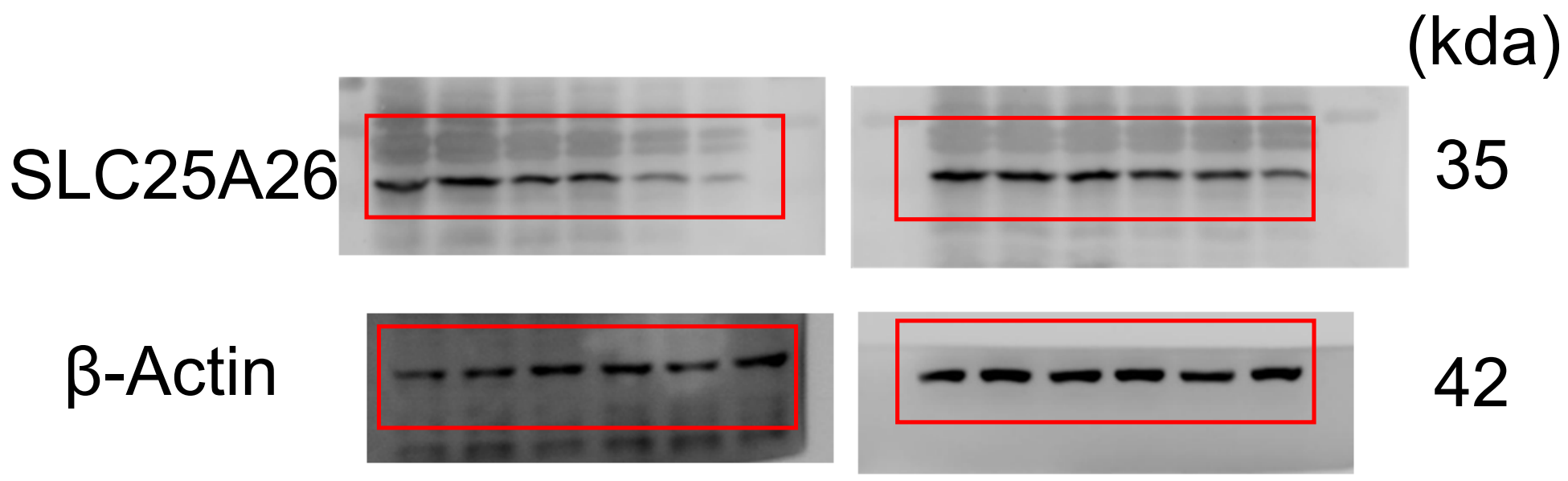


**Fig. 4F**

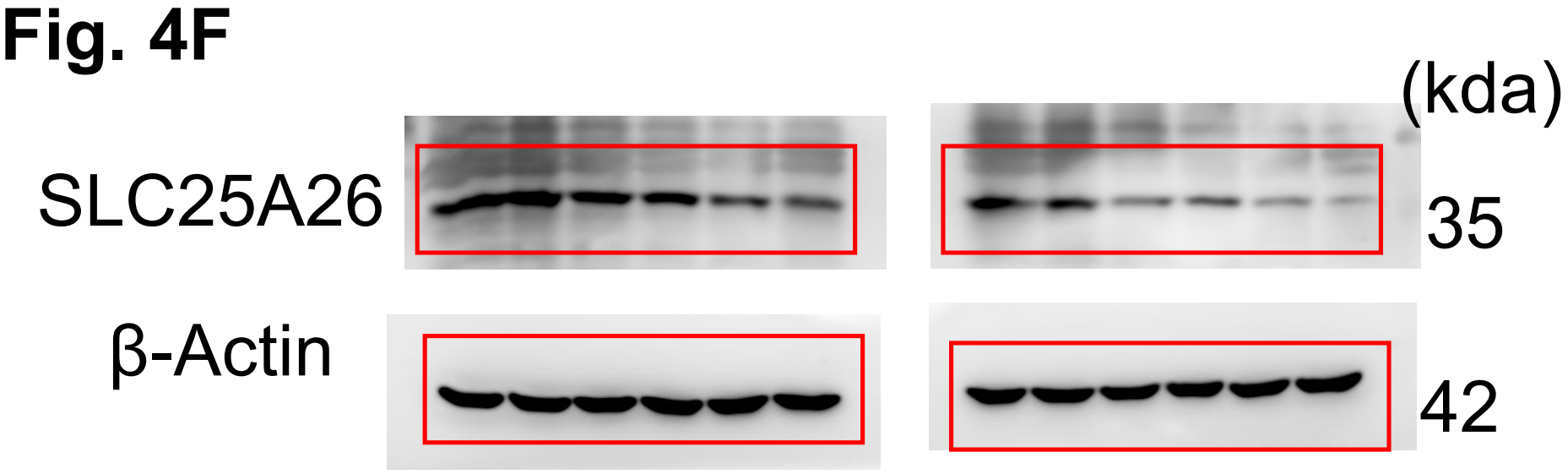

Fig. 4H

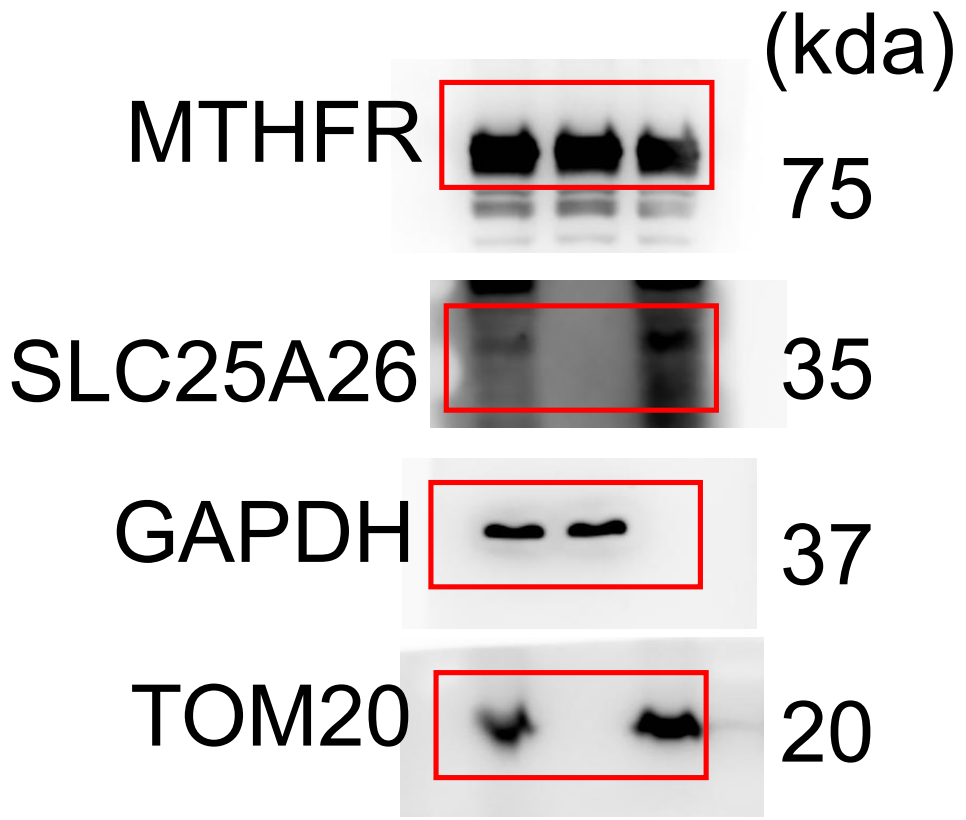


Fig. 4I

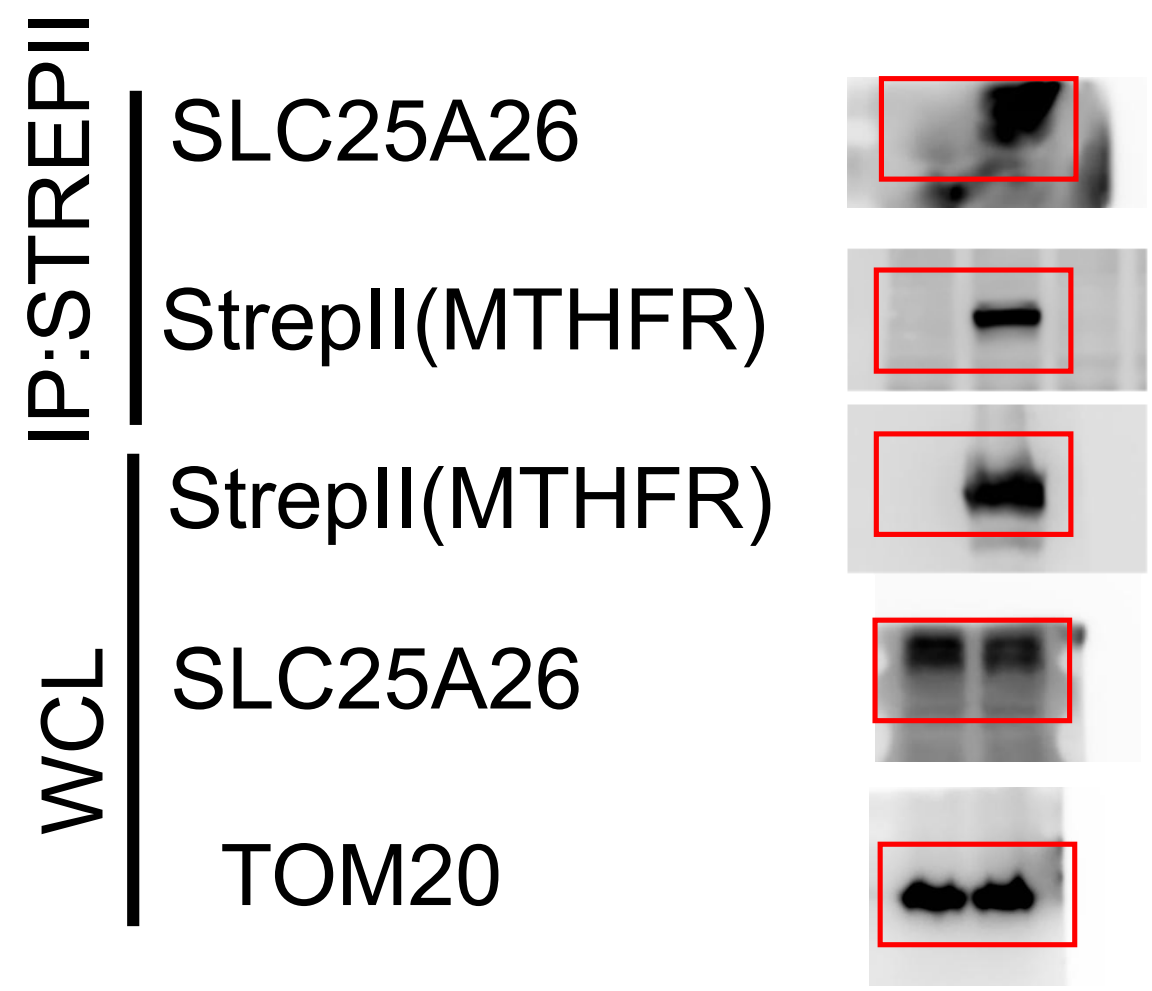


Fig. 4J

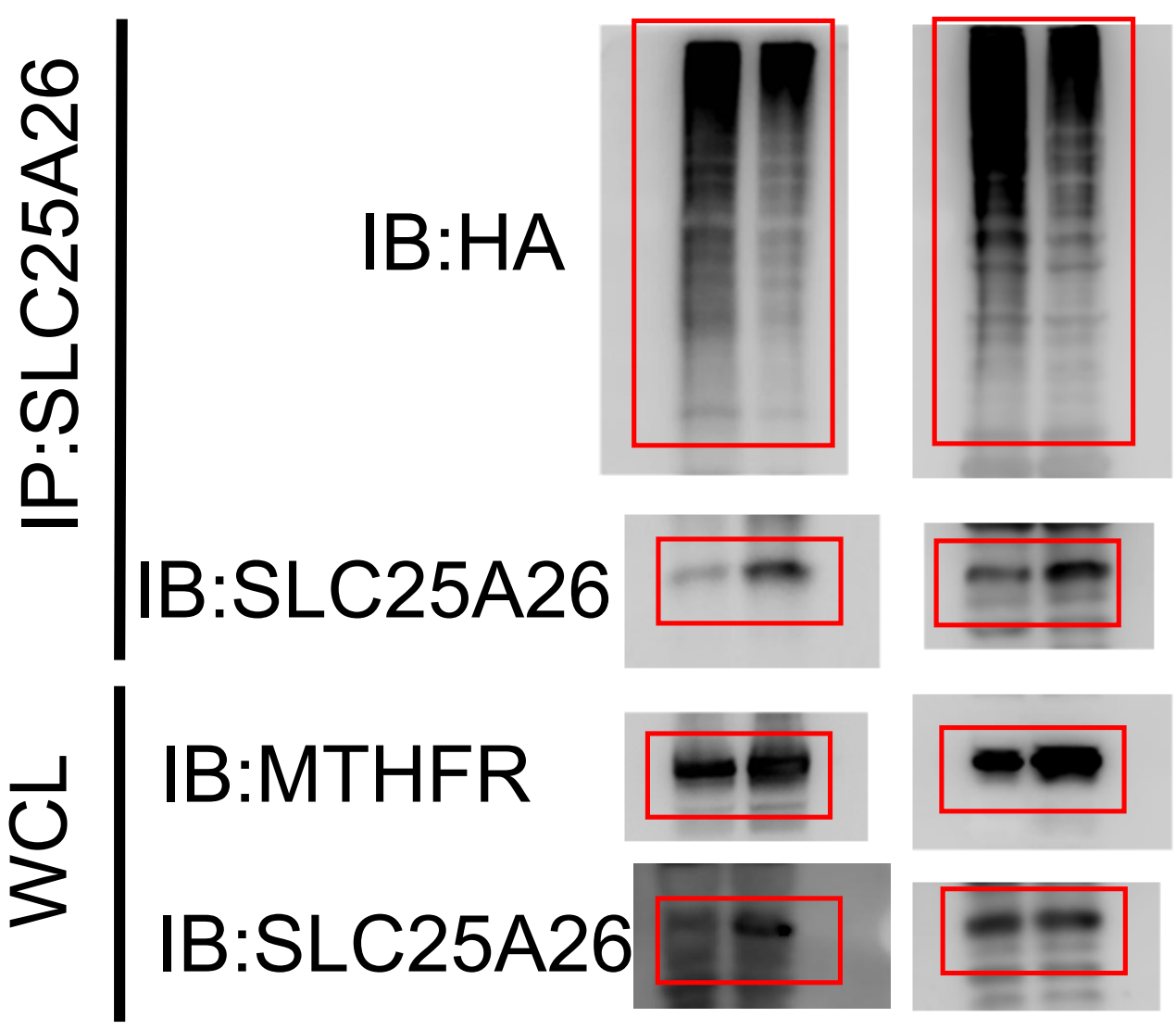


Fig. 4K

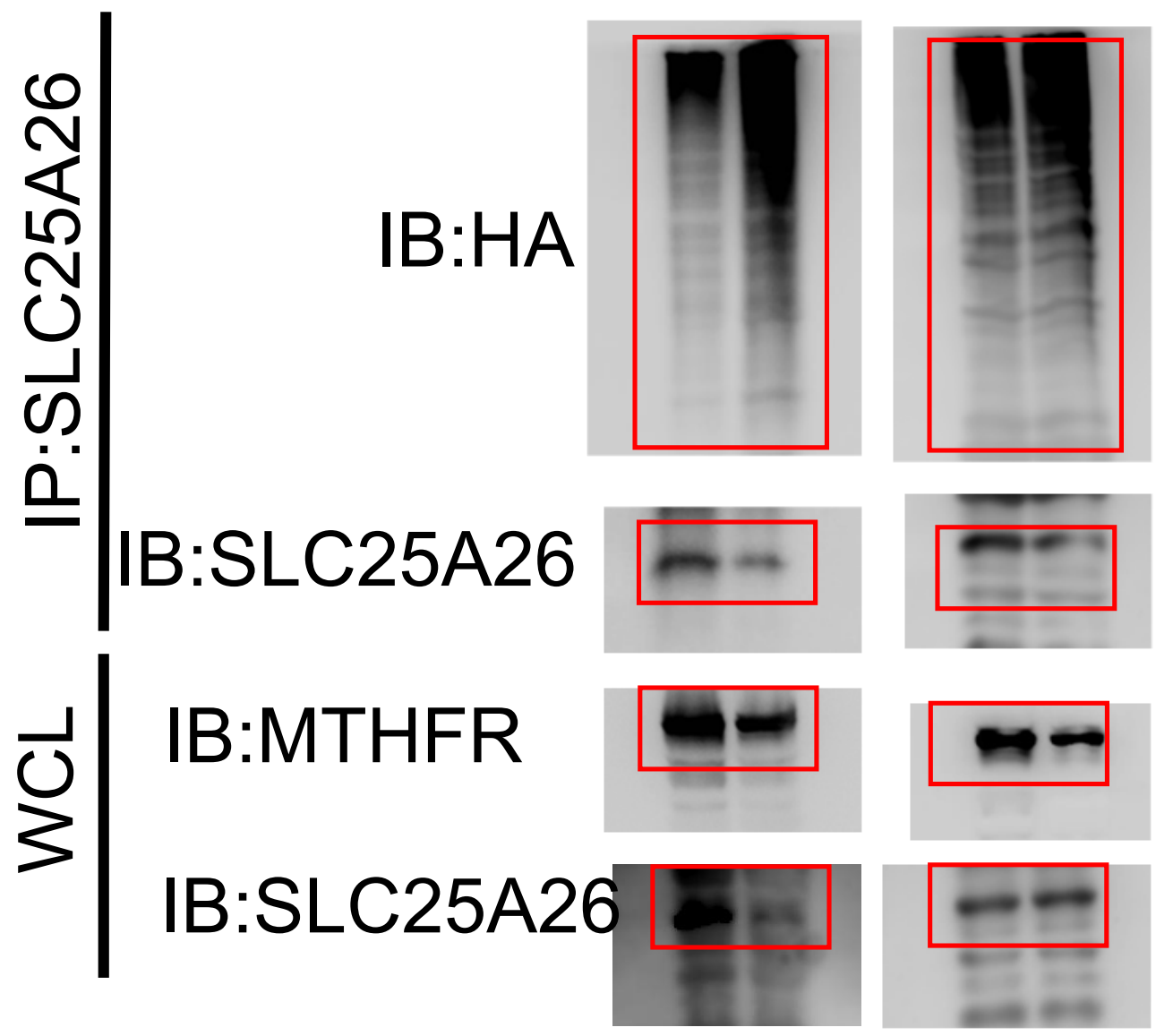

**Fig. 4N**

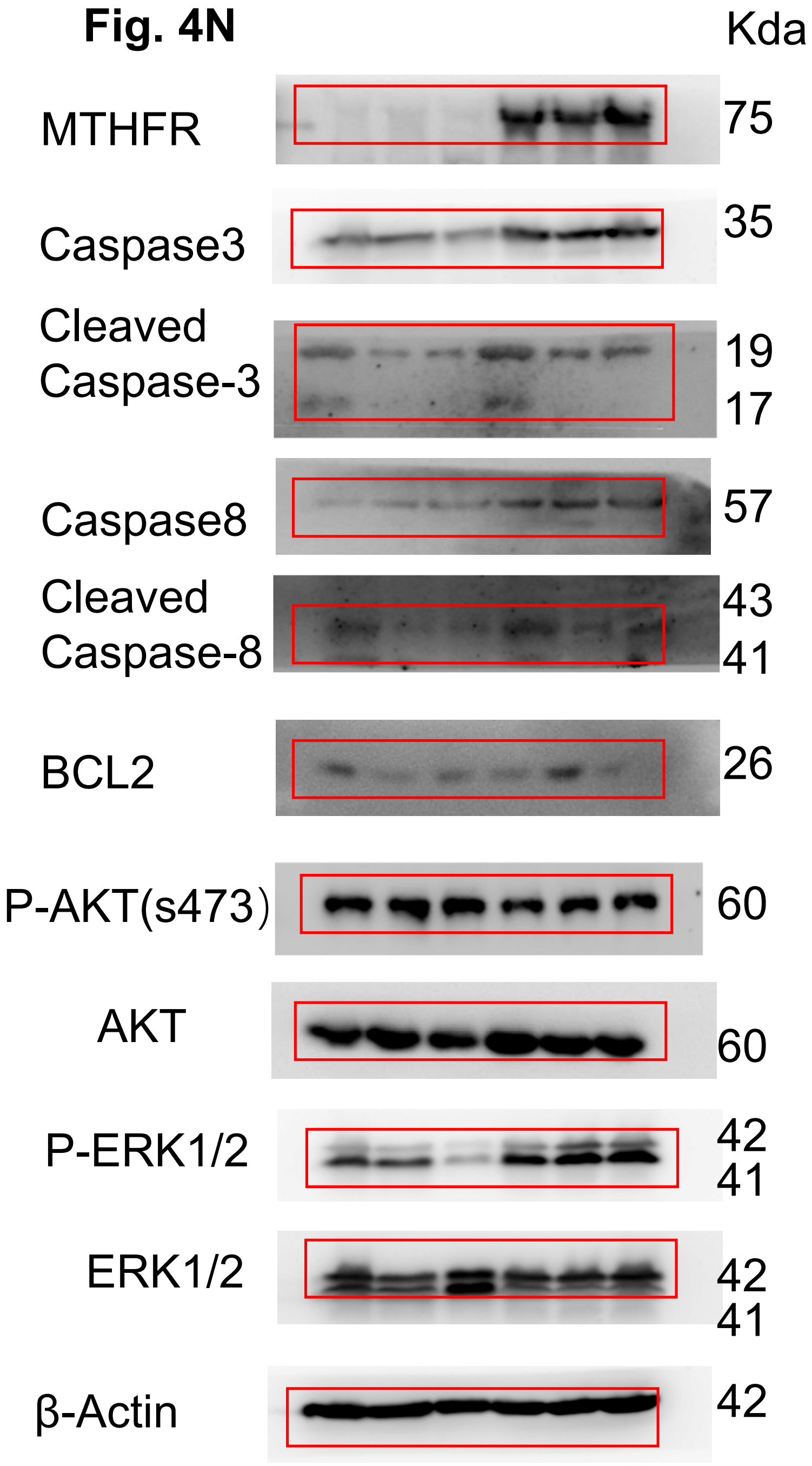
Kda
MTHFR
75
Caspase3
35
Cleaved Caspase-3
19
17
Caspase8
57
Cleaved Caspase-8
43
41
BCL2
26
P-AKT(s473)
60
AKT
60
P-ERK1/2
42
41
ERK1/2
42
41
β-Actin
42

**Fig. 5C**

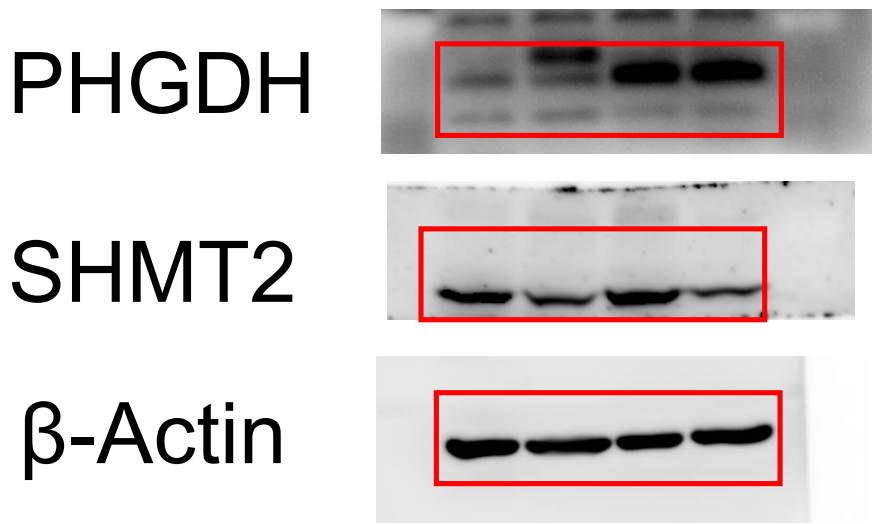
PHGDH
SHMT2
β-Actin

**Fig. 5I**

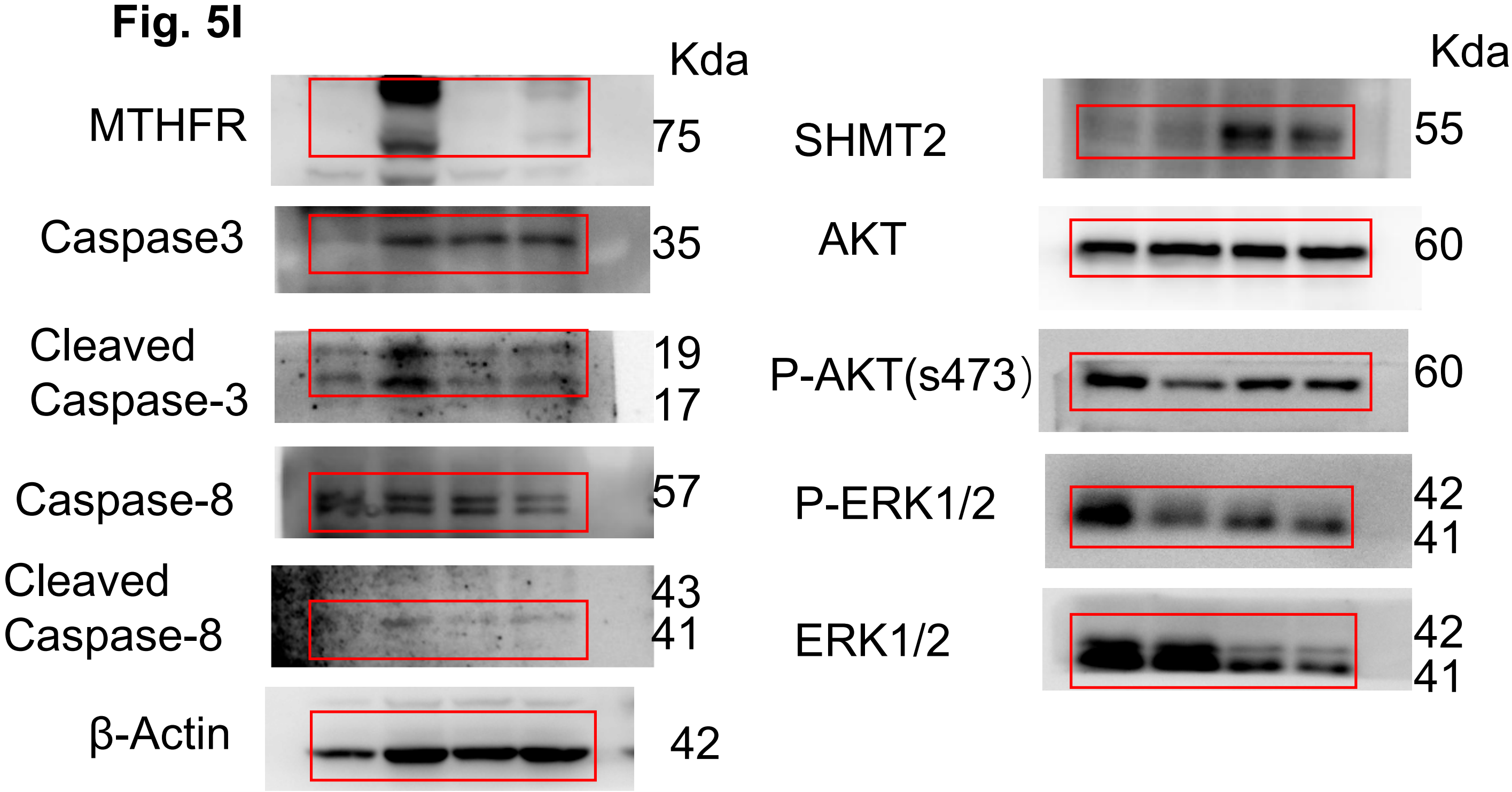
Kda
MTHFR
75
Caspase3
35
Cleaved
Caspase-3
19
17
Caspase-8
57
Cleaved
Caspase-8
43
41
β-Actin
42
Kda
SHMT2
55
AKT
60
P-AKT(s473)
60
P-ERK1/2
42
41
ERK1/2
42
41

**Fig. 6C**

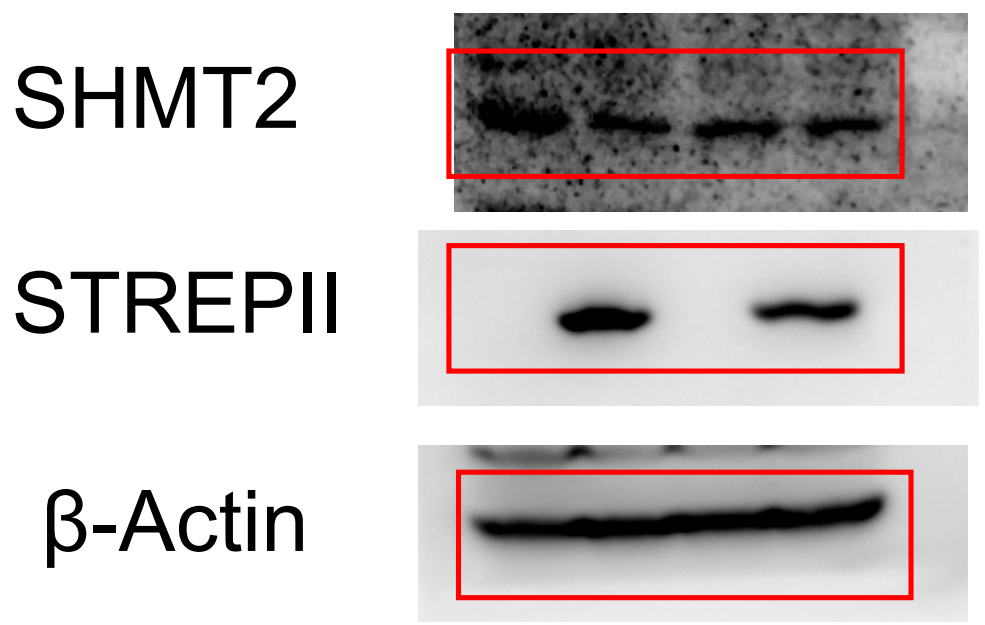


**Fig. 6D**

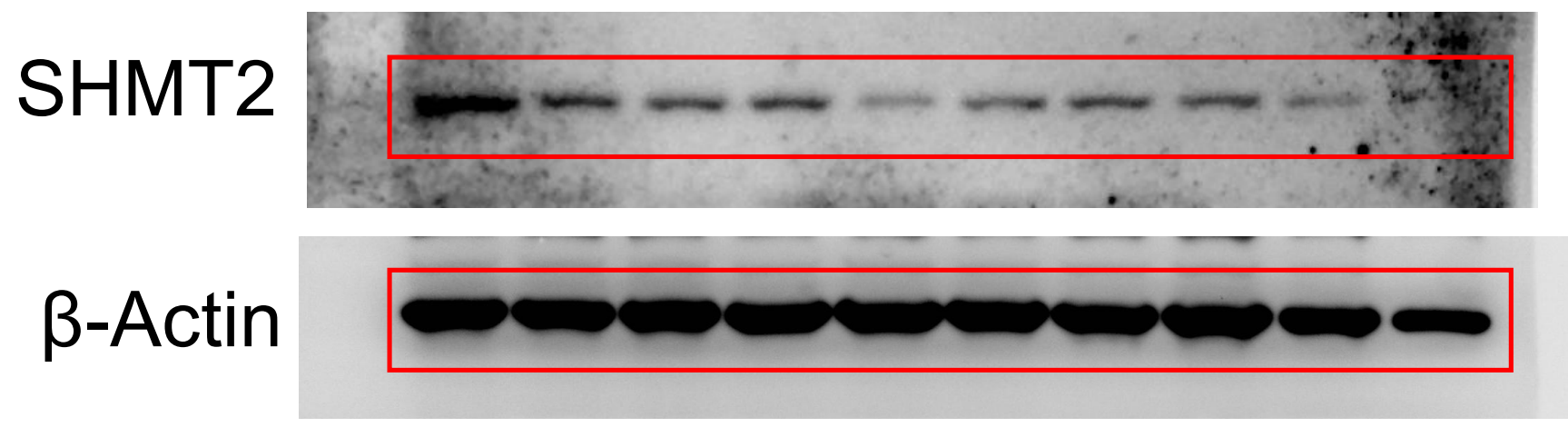


**Fig. 6F**

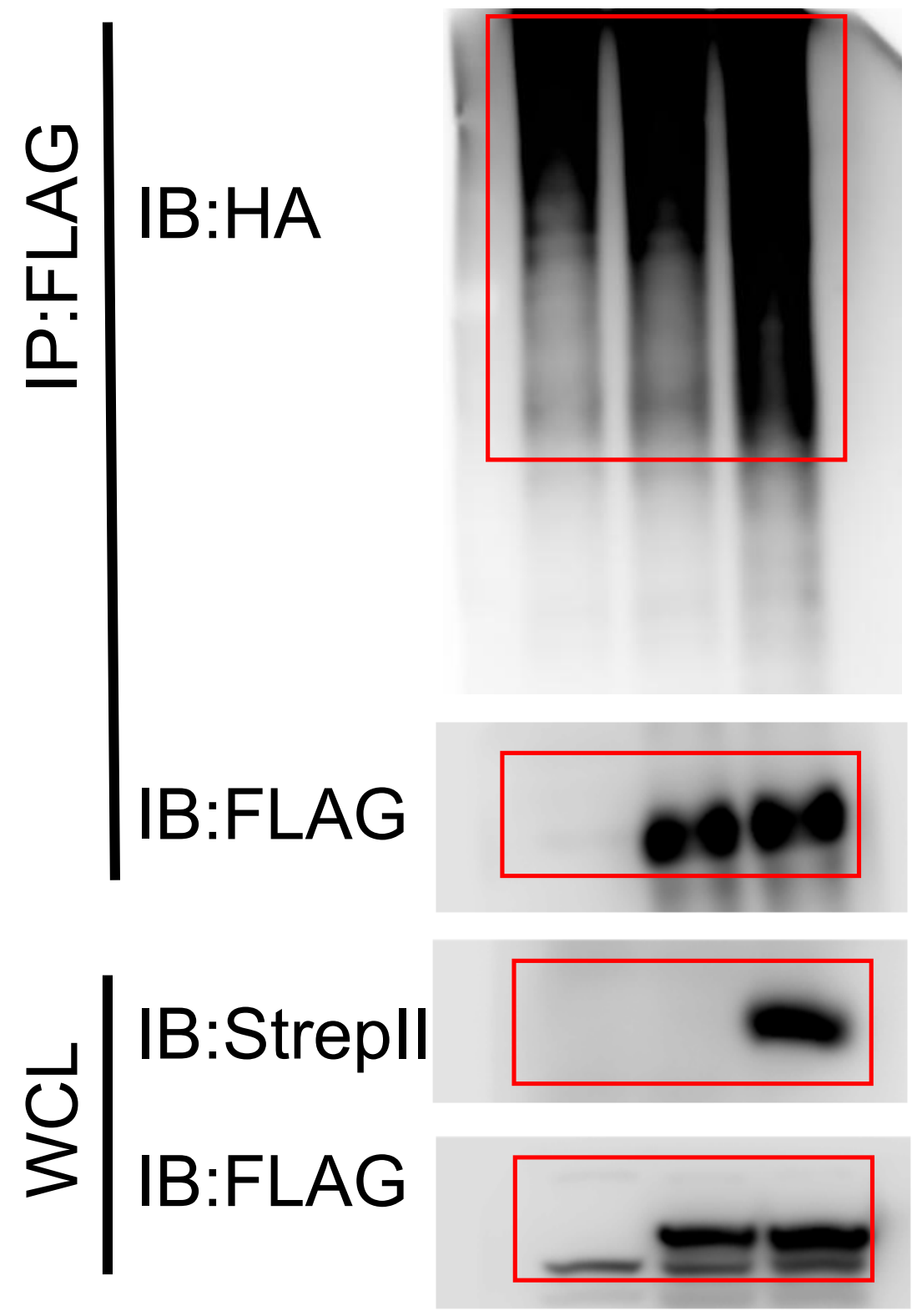


**Fig. 6G**

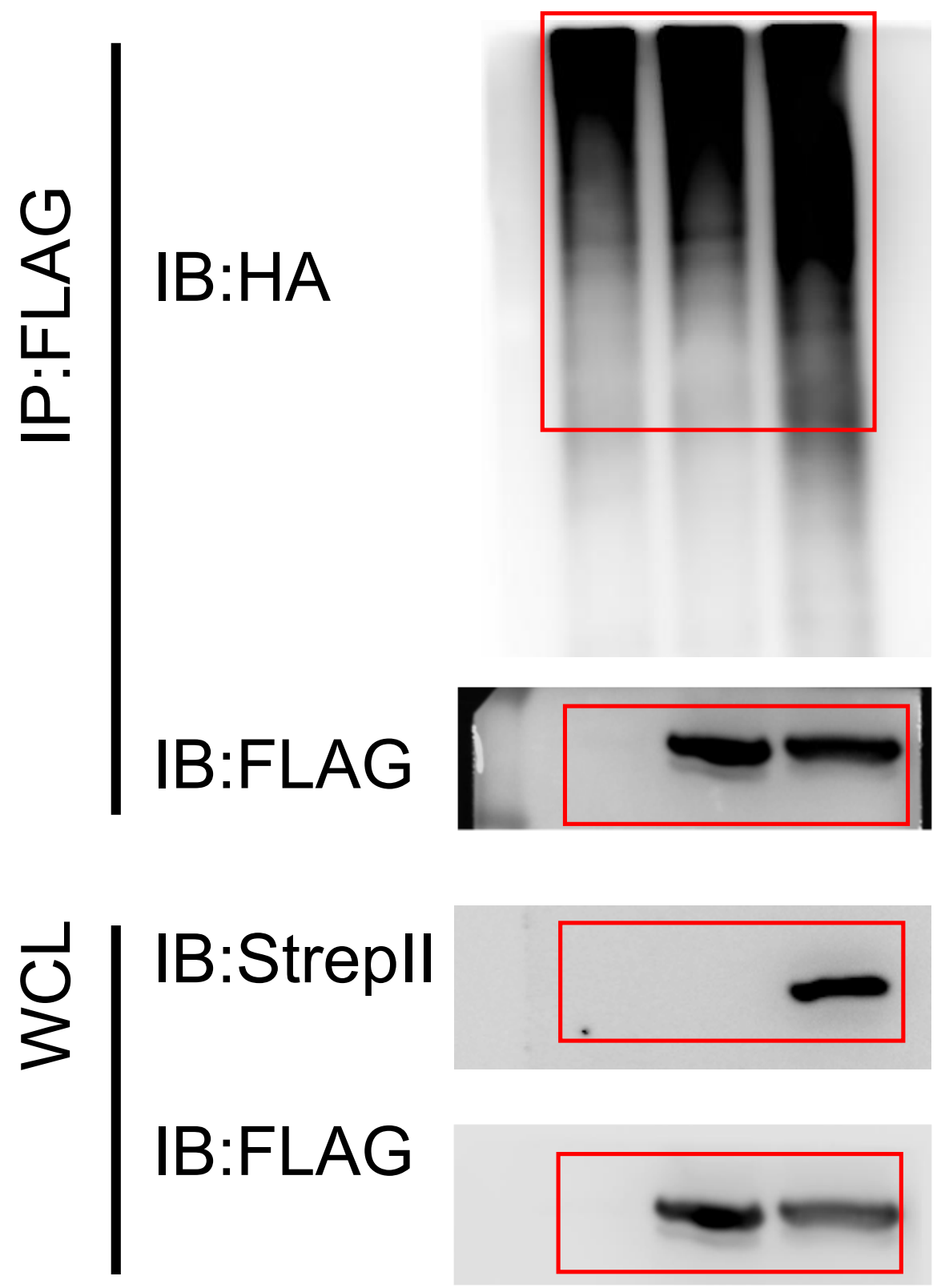


**Fig. 6L**

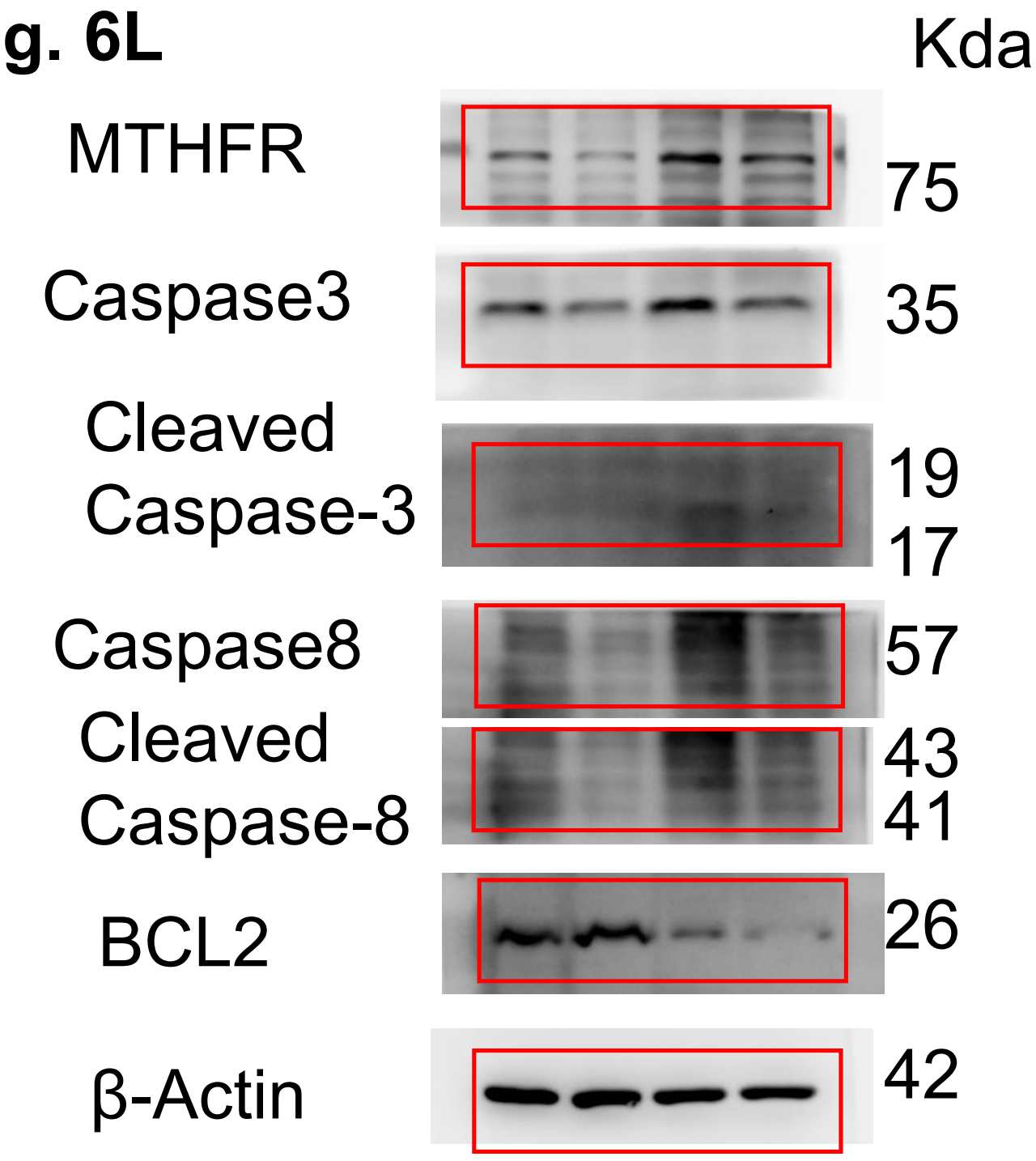

**Fig. S3B**

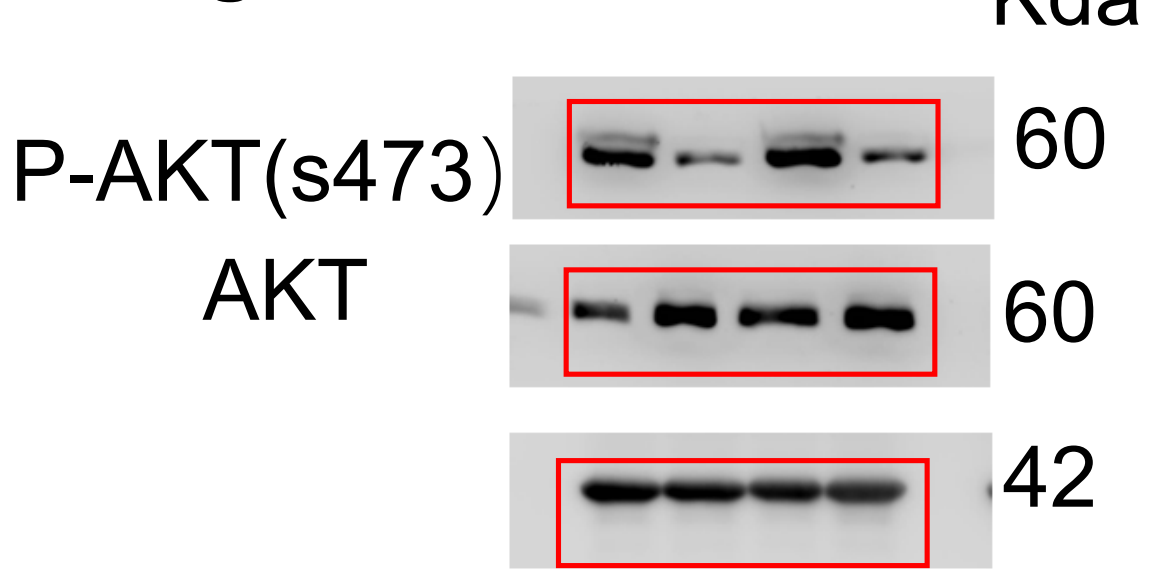


**Fig. S3C**

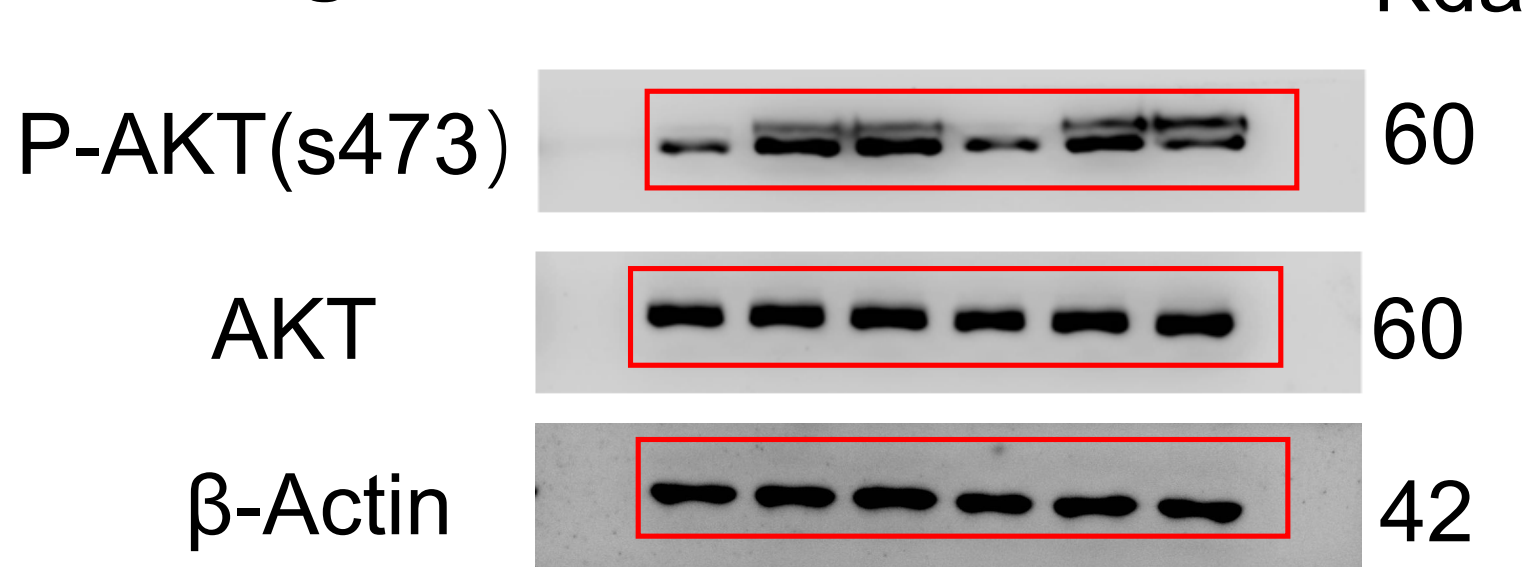


**Fig. S4E**

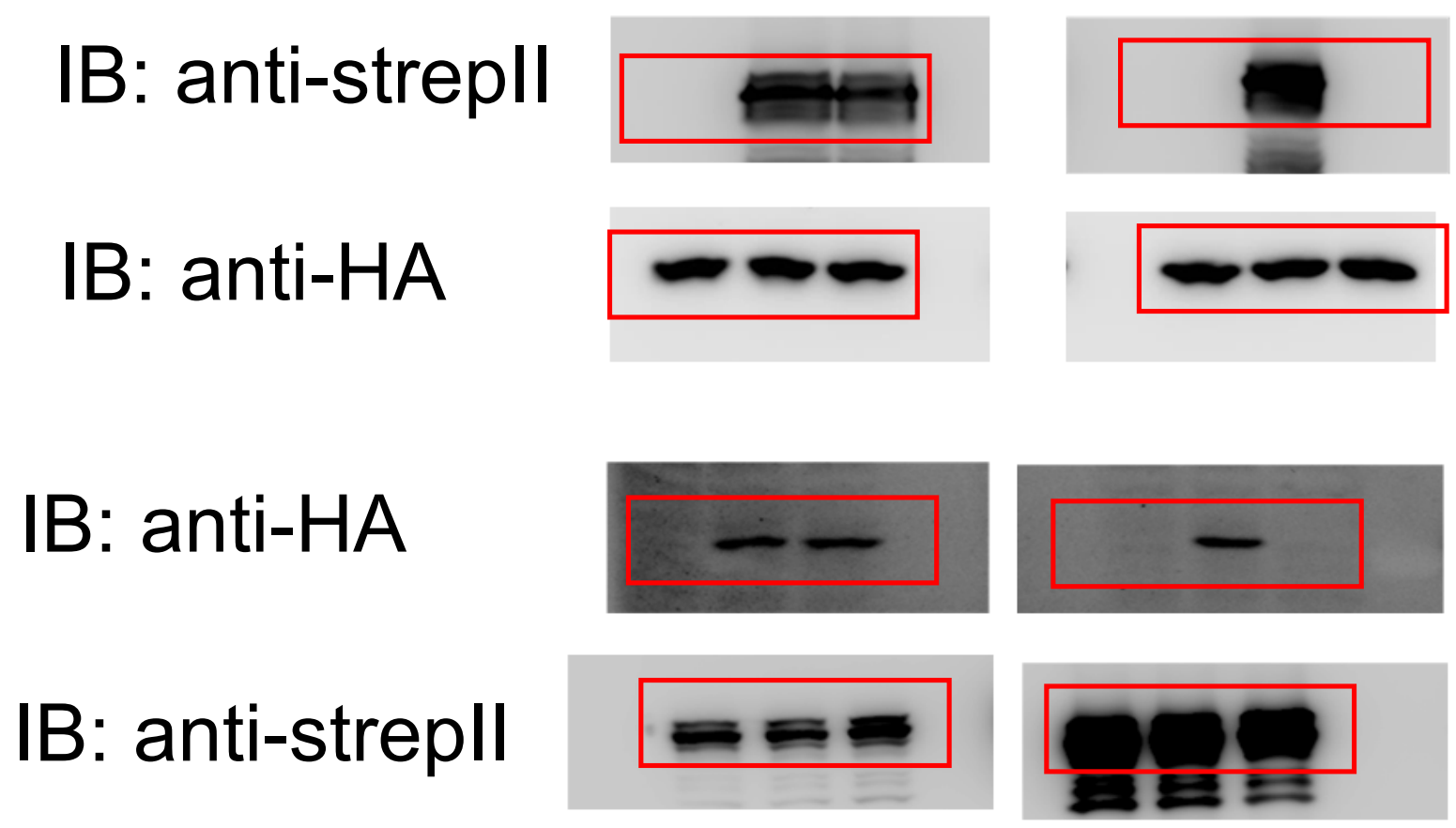


**Fig. S5F**

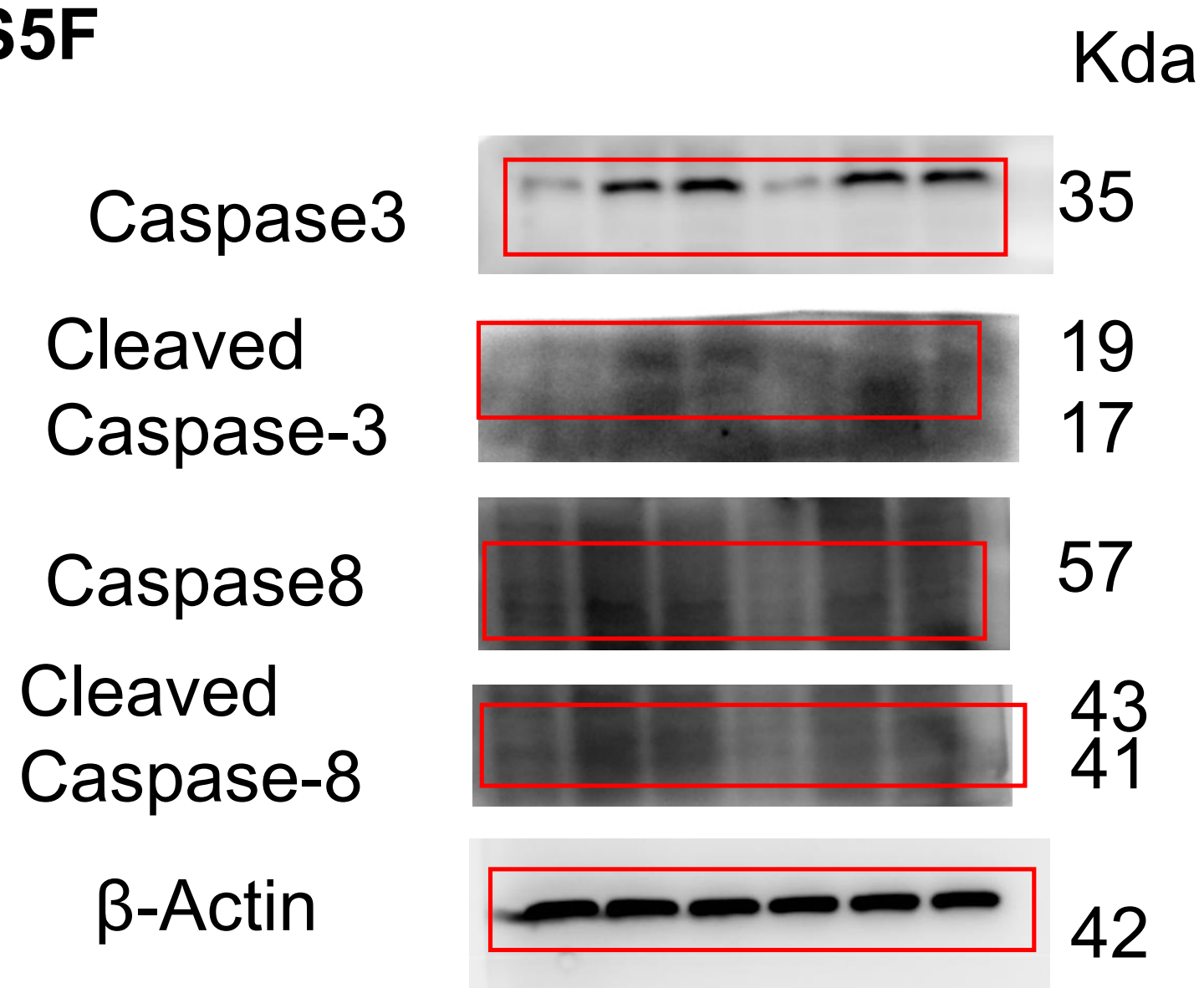

Supplement: Supplementary file 2 [file 41419_2026_8874_MOESM2_ESM.pdf]
